# Unveiling Buried Air Gaps and Interface Inhomogeneities in van der Waals Stacks via Microreflectance Mapping for Twistoptics Applications

**DOI:** 10.1021/acsanm.6c02110

**Published:** 2026-08-18

**Authors:** Julia G. Prieto, Adam Roselló, Abel Martínez-Suárez, Enrique Terán-García, Christian Lanza, Yolanda Álvarez, Stephan Kasemann, Stefan Partel, Kenji Watanabe, Takashi Taniguchi, Adrián Fernández-Gavela, Dimas G. de Oteyza, Luis Javier Martínez, Aitana Tarazaga Martín-Luengo, Pablo Alonso-González, Javier Martín-Sánchez

**Affiliations:** † Department of Physics, 530975 University of Oviedo, Oviedo 33006, Spain; ‡ Nanomaterials and Nanotechnology Research Center (CINN), CSIC-University of Oviedo-Principality of Asturias, Avenida de La Vega 4-6 El Entrego, San Martin Del Rey Aurelio, 33940 Asturias, Spain; § Donostia International Physics Center, 20018 San Sebastian, Spain; ∥ Research Centre for Microtechnology, FHV—Vorarlberg University of Applied Sciences, Campus V, Hochschulstraße 1, 6850 Dornbirn, Austria; ⊥ Research Center for Electronic and Optical Materials, 52747 National Institute for Materials Science, 1-1 Namiki, Tsukuba 305-0044, Japan; # Research Center for Materials Nanoarchitectonics, National Institute for Materials Science, 1-1 Namiki, Tsukuba 305-0044, Japan

**Keywords:** van der Waals materials, heterostructures, polaritonics, microreflectance, h-BN, α-MoO_3_

## Abstract

Van der Waals (vdW) heterostructures provide a versatile platform for multifunctional photonic and optoelectronic devices, yet their performance is often limited by interfacial inhomogeneities that perturb the local dielectric environment. This is particularly critical in polaritonic heterostructures, where the near-field optical response is extremely sensitive to buried defects. Here, we present a high-resolution microreflectance mapping technique for assessing interfacial quality in vdW heterostructures and enabling the fabrication of twisted polaritonic stacks. By combining hyperspectral imaging with transfer-matrix-method (TMM) fitting, we determine flake thickness directly on PDMS stamps and quantify buried air gaps and other interfacial nonuniformities in assembled stacks. Using h-BN and α-MoO_3_ heterostructures as benchmark systems, we detect buried air gaps down to 7 nm with ∼1 μm lateral resolution. Correlation with scattering-type scanning near-field optical microscopy (s-SNOM) confirms the quantitative accuracy of the method and shows that nanometric air gaps already produce measurable changes in phonon-polariton (PhP) propagation. Our results establish microreflectance mapping as an accessible far-field approach for quantitative interfacial control in polaritonic heterostructures, a key requirement for twistoptics applications. To facilitate implementation, we provide the analysis in an open-source graphical user interface that can be integrated into standard optical-microscope setups.

## Graphical abstract

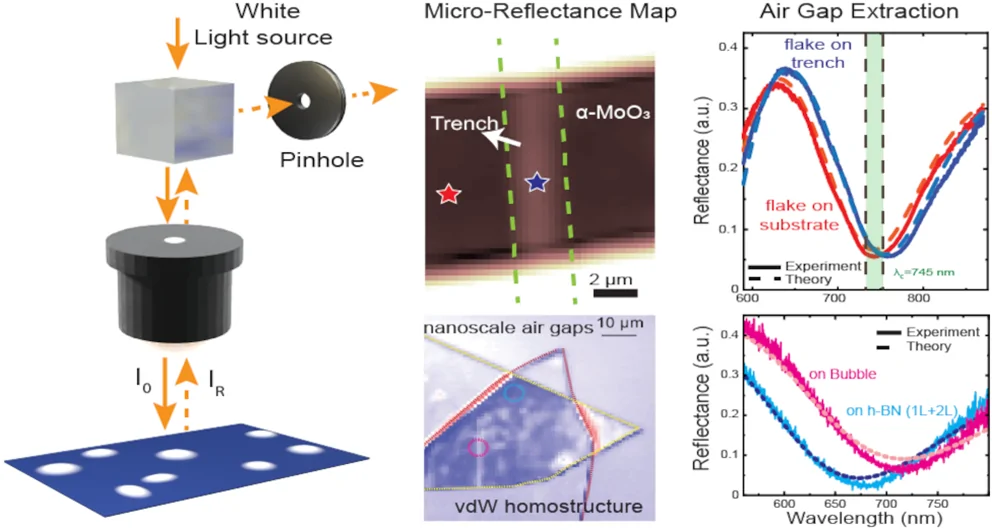

## 1 Introduction

van der Waals (vdW) materials have attracted considerable attention due to their outstanding tunable physical properties, enabling a wide range of applications,^1,2^ including ultrathin field-effect transistors based on two-dimensional semiconductors,^3,4^ optoelectronic devices such as photodetectors,^5^ and biosensing platforms based on vdW heterostructures.^6^ Beyond these applications, polar vdW materials have emerged as promising platforms for nano-optics, where strong light-matter coupling enables subwavelength confinement of light^7^ via surface electromagnetic waves. These waves, known as polaritons, result from the coupling between light and collective matter excitations.^8–10^ A prominent example of such hybrid excitations are phonon-polaritons (PhPs) in polar vdW crystals, which display strong electromagnetic field confinement and low-loss propagation at the nanoscale.^9,11,12^ Interestingly, stacking different vdW crystals into vertical homo- and heterostructures provides an additional degree of freedom to engineer polaritonic responses of the assembly. Specifically, the PhPs dispersion can be tuned to induce transitions from open to closed isofrequency curves, (IFCs)—constant-frequency slices of the dispersion surface in momentum space^11,13^ —, by tailoring the number, material and thickness of the constituent layers, as well as their relative twist angle. The twist-dependent control of light-matter interaction, often referred to as twistoptics,^14^ has revolutionized the field of nano-optics, offering an unprecedented degree of control over light propagation at the nanoscale.^15–20^ A paradigmatic example is the canalization of PhPs in twisted stacks of anisotropic materials like α-MoO_3_,^12,21^ characterized by unidirectional propagation at the nanoscale.^22–24^

In vdW heterostructures, the optical and polaritonic response is strongly governed by the nanoscale structure of buried interfaces, where nanometric air gaps, bubbles, or partial delamination between flakes can significantly alter interlayer coupling, thus modifying the local dielectric environment and reshaping the polaritonic dispersion.^25^ For instance, the wavelength of PhPs in single polar dielectric slabs scales directly with the slab thickness.^26^ This strong dependence on both the thickness of the constituent layers and the quality of the interfaces makes the experimental realization of high-quality twistoptics devices particularly challenging. In particular, the assembly of twisted vdW stacks requires selecting single flakes with specific thicknesses from the inherently random distribution produced by micromechanical exfoliation of bulk crystals onto a polymer stamp (e.g., polydimethilsiloxane, PDMS). The selected flakes are then deterministically dry-transferred onto target substrates, after which it is necessary to assess the presence of nanometer-scale air gaps or organic residues, which are usually introduced in the stacking process.^27–29^ Because efficient near-field coupling between layers is central in twistoptics, these buried inhomogeneities can act as scattering centers or dielectric barriers, severely affecting the polaritonic propagation and altering the expected dispersion relations. Similar sensitivity to interfacial nanogaps has been reported in other evanescent-field-mediated processes, such as near-field radiative heat transfer between twisted vdW homo- and heterostructures, where nanometric separations between layers strongly modulate the electromagnetic coupling and energy transfer rates.^30^

However, current characterization methods remain a bottleneck in this multiple-step fabrication process. Atomic Force Microscopy (AFM), the standard technique for thickness determination, faces severe limitations: it is experimentally cumbersome and cannot be reliably performed on the soft viscoelastic PDMS polymers used for deterministic dry transfer. This forces the transfer of flakes onto hard intermediate substrates (like SiO_2_/Si) solely for AFM measurement, only to pick them up again for the final heterostructure assembly, which is a multistep process that increases the risk of contamination and vdWs crystals surface damage. Furthermore, while AFM excels at surface topography, it is very limited to reveal buried inhomogeneities,^31,32^ since it cannot differentiate between a pristine interface and one containing a uniform air gap. Ultimately, this leads to wrong thickness estimations and potentially unexplained polariton propagation properties in the final polaritonic device. While transmission electron microscopy (TEM) can reveal these gaps,^27^ it is a destructive technique incompatible with rapid device fabrication. Moreover, samples for TEM analysis require specific preparation and are typically fabricated exclusively for that characterization, meaning that the flakes examined by TEM are not the same ones ultimately used in functional devices.

Optical characterization offers a promising nondestructive alternative due to its simplicity and relatively fast acquisition. Several optical techniques such as Raman spectroscopy, photoluminescence, optical absorption and contrast have been used for the identification of the number of layers in few-layer vdW transition metal dichalcogenide (TMD) and graphene flakes.^33,34^ In addition, microreflectance/transmittance spectroscopy has been established as a versatile tool for layer identification and optical characterization of 2D materials,^33^ while TMM-based analysis of reflectance spectra has been successfully applied to thickness determination in single vdW flakes such as InSe, hBN, and MoO_3_ on SiO_2_/Si substrates,^35–37^ to composition and layer-number determination in graphene/hBN heterostructures,^38^ and to thickness evaluation of birefringent or encapsulated vdW layers.^39^ However, a robust fabrication-oriented procedure capable of combining two critical capabilities is still lacking. First, the precise determination of flake thickness directly on the soft and optically transparent polymer stamps used for deterministic dry transfer, and second, the spatially resolved imaging and quantification of buried interfacial inhomogeneities in assembled vdW heterostructures. Such a capability is particularly relevant for polaritonic vdW materials, where nanometric air gaps, bubbles, or other interfacial discontinuities directly modify the local dielectric environment and can therefore strongly affect phonon-polariton propagation. Addressing this need would enable thickness measurements prior to assembly, simplifying the fabrication workflow, while also allowing the identification and quantification of interfacial air gaps once the stacks are assembled on the final substrates.

Here, we present a high-resolution and versatile microreflectance mapping technique for assessing interfacial quality in vdW heterostructures, providing a practical tool for the fabrication of twisted polaritonic stacks. First, we combine high-resolution hyperspectral imaging with a TMM fitting algorithm to (i) determine flake thickness directly on commercially available PDMS stamps, eliminating the need for intermediate substrates, and both (ii) image and quantify the thickness of buried air gaps and other interfacial nonuniformities in assembled stacks. Second, we validate the method on polaritonic heterostructures by correlating microreflectance hyperspectral maps with near-field s-SNOM measurements. We further show that nanometric air gaps as small as 7 nm already have a measurable impact on PhPs propagation in well-controlled reference heterostructures consisting of nanometer-thick α-MoO_3_ flakes on patterned Si substrates, underscoring both the need for high-resolution detection of interfacial air gaps for applications in polaritonics and the accuracy of our approach. This methodology not only streamlines the fabrication of twisted heterostructures by enabling in situ flake selection during the fabrication process but also provides quantitative control over interfacial quality. Such control is not achievable with conventional probe microscopies, yet is crucial for ensuring the optical parameters in polaritonic heterostructures for twistoptics applications. To facilitate its implementation, the analysis is incorporated into an open-source graphical user interface that can be directly integrated into standard optical-microscope setups.

## 2 Experimental Section

### 2.1 Fabrication of Si Trenches

A positive photoresist (SPR 955-CM 0.7) was spin-coated onto 100 mm Si substrates using a SUSS ASC200 spin coater, resulting in a resist thickness of approximately 560 nm. Patterns were defined by direct write laser lithography using a DWL66+ system (Heidelberg Instruments) with a 2 mm write head. After exposure, the samples were post exposure baked at 115 °C for 120 s and developed for 80 s in a tetramethylammonium hydroxide (TMAH) based developer (AZ 726 MIF), followed by rinsing in deionized water and drying under nitrogen flow. Residual resist was removed by a short oxygen plasma descum (10 s) using an AMS100 reactive ion etching system (Adixen). Si etching was subsequently performed by reactive ion etching using the same Adixen AMS100 Tool. For etch depths in the range of 500 nm, a fast gas chopping process was used with SF_6_ and C_4_F_8_ + O2 gas chemistry at a temperature of 10 °C with 25 s (10 chopping cycles) etch time. For lower etch depths in the range of 100 nm, a continuous RIE process was used with C_4_F_8_ + He + CH4 gas chemistry at a temperature of −10 °C. In this latter case, the etch time was adjusted according to the target etch depth (for example, 62 s for 100 nm and 93 s for 150 nm). After etching, the remaining photoresist was removed by oxygen plasma ashing using the AMS100 system (Adixen).

### 2.2 h-BN and α-MoO_3_ Samples Preparation

Thick crystals of h-BN (provided by Prof. Kenji Watanabe and Prof. Takashi Taniguchi) and commercial α-MoO_3_ (HQ Graphene) were first peeled off from bulk materials and thinned down using Nitto tape. Next, the exfoliated thin 2D crystals were transferred on PDMS-Polyester commercial stamps (WF Film 4′ 6 mils X4 from Gel-Pak). Flakes with thicknesses ranging from 10 to over 400 nm were considered for calibration and validation of the refractive index of h-BN and α-MoO_3_, while homogeneous pieces of both materials between 100 and 400 nm were selected for studying interfaces via microreflectance maps. All flakes were transferred to Si substrates using the dry transfer technique.

### 2.3 AFM Measurements

The thickness of thin single slabs of h-BN and α-MoO_3_ was measured in a scattering-type scanning near-field optical microscope (s-SNOM) from Neaspec in AFM mode. All measurements employed Si tips in tapping mode, with a tapping frequency of Ω ∼ 285 kHz and an oscillation amplitude of around 200 nm.

### 2.4 Microreflectance Setup for Refractive Index Extraction and In Situ Thickness Estimation in 2D Crystals

Experimental reflectance of h-BN and α-MoO_3_ single slabs was measured using a modified optical microscope as described in ref 33, based on a Leica DM 2700 M optical microscope in reflection mode using the objective N Plan 20×, NA = 0.4. The lighting was provided by an unpolarized white light source (Thorlabs broadband Halogen lamp OSL2) emitting in the range from 400 to 900 nm and which was normally incident on the sample. The experimental reflectance was measured using a fiber-coupled compact CCD spectrometer (Thorlabs CCS200/M). The multimode fiber, with a core of 200 μm and NA = 0.22, served as a pinhole, allowing for a spatial resolution of about 10 μm for the 20X objective.

### 2.5 Microreflectance Mapping

Reflectance imaging measurements were performed in an adapted microphotoluminescence (micro-PL) system, with a 100×, NA = 0.7 objective (Mitutoyo), using an unpolarized white light source (Thorlabs broadband Halogen lamp OSL2). Spatial maps with a step resolution of 500 nm or 1 μm were obtained using an FSM-300–01 fast steering mirror combined with a 4*f* lens system (*f* = 25 cm, 2-in. diameter). The steering mirror scans the angle of the incident beam at the objective entrance pupil, while the 4f configuration relays the white light beam between the mirror and the objective, converting angular deflections into lateral displacements of the focused spot and enabling XY scanning of the sample. The reflected beam was collected by the same objective and focused onto a 25 μm pinhole using a 75 mm focal-length lens, ensuring optimal spatial resolution while suppressing out-of-focus background. The pinhole size was chosen to match one Airy unit, enabling the system to achieve the maximum theoretical lateral resolution, given by $d_{\mathrm{xy}}=\frac{1.22\lambda }{2\mathrm{NA}}≃600\;\mathrm{nm}$ for a 700 nm incident light. The lateral resolution of the maps is therefore limited by the diffraction-limited point-spread function of the focused spot, rather than by the scan step size alone. The 1 μm step size used here provides adequate sampling of micrometre-scale interfacial features while keeping acquisition times on the order of a few minutes (typical maps were acquired with an integration time of 0.1 s per pixel). The light was then coupled into a 105 μm, 0.1 NA multimode fiber and subsequently focused onto a Kymera 328i spectrometer (Andor) equipped with a Newton CCD. The microreflectance maps are obtained by integrating the reflectance signal over a selected spectral window for each pixel. This window is chosen to enhance the contrast of the features of interest but does not affect the quantitative analysis.

### 2.6 Transfer Matrix Method Numerical Simulations

Experimental reflectance spectra were fitted to the theoretical transfer matrix method using nonlinear least-squares (curve_fit function from SciPy). The model accounts for multilayer interference and depends on both the layer thicknesses and complex refractive indices. For refractive index extraction, the AFM-measured thickness of the flake was used as input, whereas for layer thickness determination, the refractive indices were taken as input. Further details of the simulations and fitting procedure are provided in the Supporting Information.

### 2.7 Scattering-Scanning Near Field Optical Microscopy

Near-field imaging measurements were carried out using a commercial scattering-type Scanning Near-Field Optical Microscope (s-SNOM) from Neaspec GmbH, featuring an Optical Parametric Oscillator (OPO) laser (model PT277-XIR-AOM-B, EKSPLA). The laser provides tunable emission from 1.4 to 18 μm via a Difference Frequency Generation (DFG) stage. Polaritonic excitations were both launched and detected using a metal-coated (Pt/Ir) atomic force microscopy (AFM) tip operated in tapping mode at a frequency of Ω ≈ 280 kHz and an oscillation amplitude of approximately 150 nm. The tip was illuminated with p-polarized infrared light from the laser, which was focused by a parabolic mirror onto the sample. The light scattered by the tip was collected by the same mirror and directed to an infrared detector (Kolmar Technologies). The detected signal, expressed as *σ_n_* = *s_n_e* ^*iϕ_n_*^, was demodulated at the third harmonic (*n* = 3) of the tip’s oscillation frequency to suppress background contributions. The amplitude (*s*_3_) and phase (ϕ_3_) components of the near-field signal were subsequently retrieved using a pseudoheterodyne interferometric detection scheme.

## 3 Results and Discussion

Our microreflectance system relies on a simple optical configuration consisting of an objective lens, a beam splitter, and a pinhole (Figure 1a). A collimated white-light beam with intensity *I*_0_ is transmitted through the beam splitter and focused onto the sample by the objective. The reflected beam returns to the beam splitter with intensity *I*_R_, and is then coupled to a spectrometer via an optical fiber that also acts as a pinhole or spatial filter (see Experimental Section), using an optical configuration similar to that reported by Frisenda et al.^33^ The lateral resolution on the flake is then determined by the objective and the fiber core diameter (see Experimental Section). This straightforward configuration can be easily incorporated into optical setups in fabrication laboratories and extended to more advanced platforms for microreflectance mapping by scanning the incident beam across the sample, as implemented in this work to obtain spatially resolved reflectance maps in vdW heterostructures, shown from Figure 2 onward.

**Figure 1:**
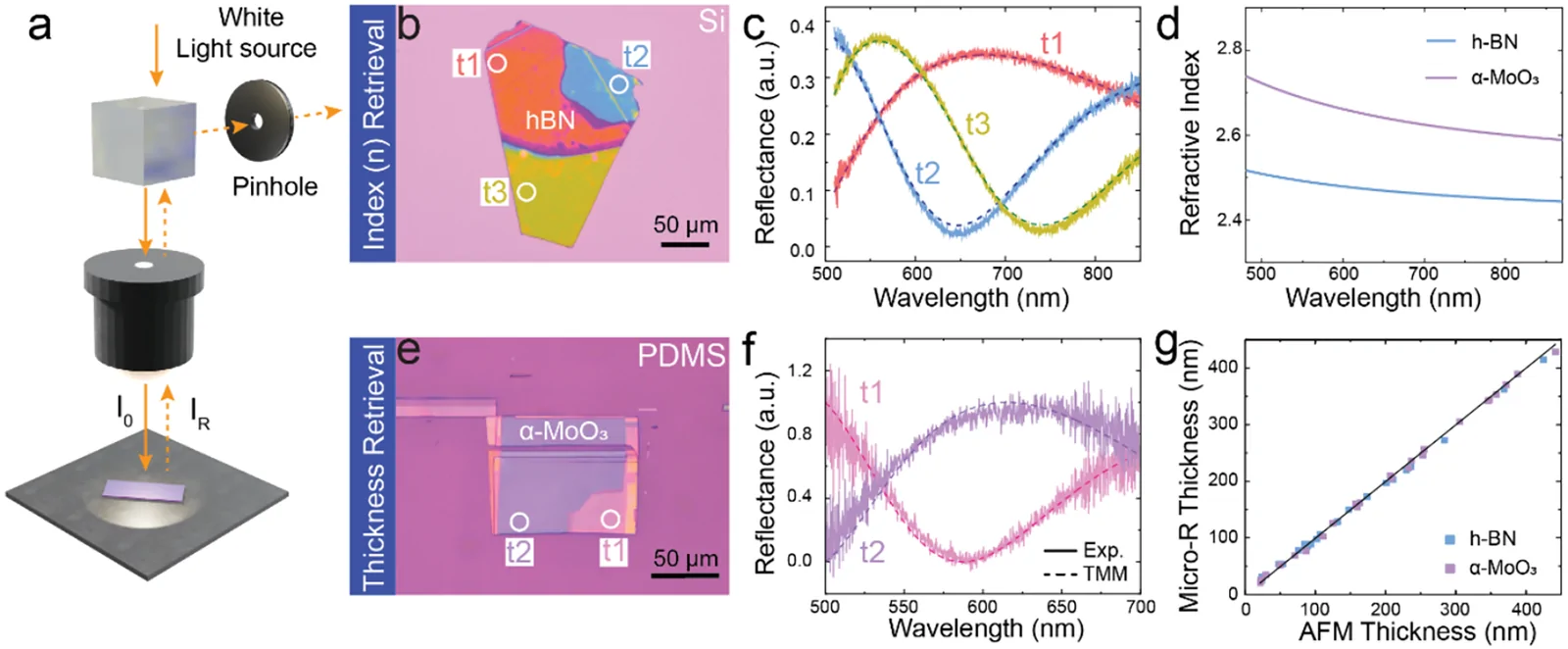
Extraction of refractive indices and validation of optical thickness determination. (a) Schematic illustration of the microreflectance setup. *I*_0_ and *I*_R_ represent the incident and reflected light intensities, respectively. The workflow consists of two stages: first, the extraction of the refractive index for h-BN and α-MoO_3_ using a calibration set of flakes on Si (b–d), and second, the validation of the obtained values through the determination of the thickness of single flakes of initially unknown thickness (e–g). (b) Optical image of a h-BN flake on a Si substrate showing three distinct thickness regions highlighted with white circles (t_1_, t_2_, t_3_). The observed different colors arise from thin-film interference in regions with different flake thicknesses under white-light illumination in the optical microscope. (c) Experimental reflectance spectra (solid lines) measured on positions (t_1_, t_2_, t_3_) in (b), together with the theoretical fits (dashed lines) based on the TMM. (d) Extracted real part of the refractive indices *n* for h-BN and α-MoO_3_ derived from the global fitting of the calibration set of flakes. The data is fitted to a Cauchy dispersion law with parameters *A* = 2.412 ± 0.002 and *B* = (2.42 ± 0.04)·10^–2^ μm^2^ for h-BN (blue curve), and *A* = 2.523 ± 0.002 and *B* = (4.98 ± 0.05)·10^–2^ μm^2^ for α-MoO_3_ (purple curve). (e) Optical image of an α-MoO_3_ flake transferred onto a PDMS-Polyester stamp showing two distinct thickness regions (different colors) highlighted with white circles (t_1_, t_2_). (f) Experimental reflectance spectra (solid lines) at positions t_1_ and t_2_ highlighted in (e) alongside their theoretical TMM fits (dashed lines). (g) Correlation plot of optically extracted thickness (*y*-axis) against the AFM-measured thickness (*x*-axis) for various h-BN and α-MoO_3_ flakes. The solid black line represents the ideal 1:1 correspondence. Linear fits yield systematic errors of 4% for h-BN (blue dots) and 1% for α-MoO_3_ (purple dots), calculated from the deviation of the fitted slope from unity.

**Figure 2:**
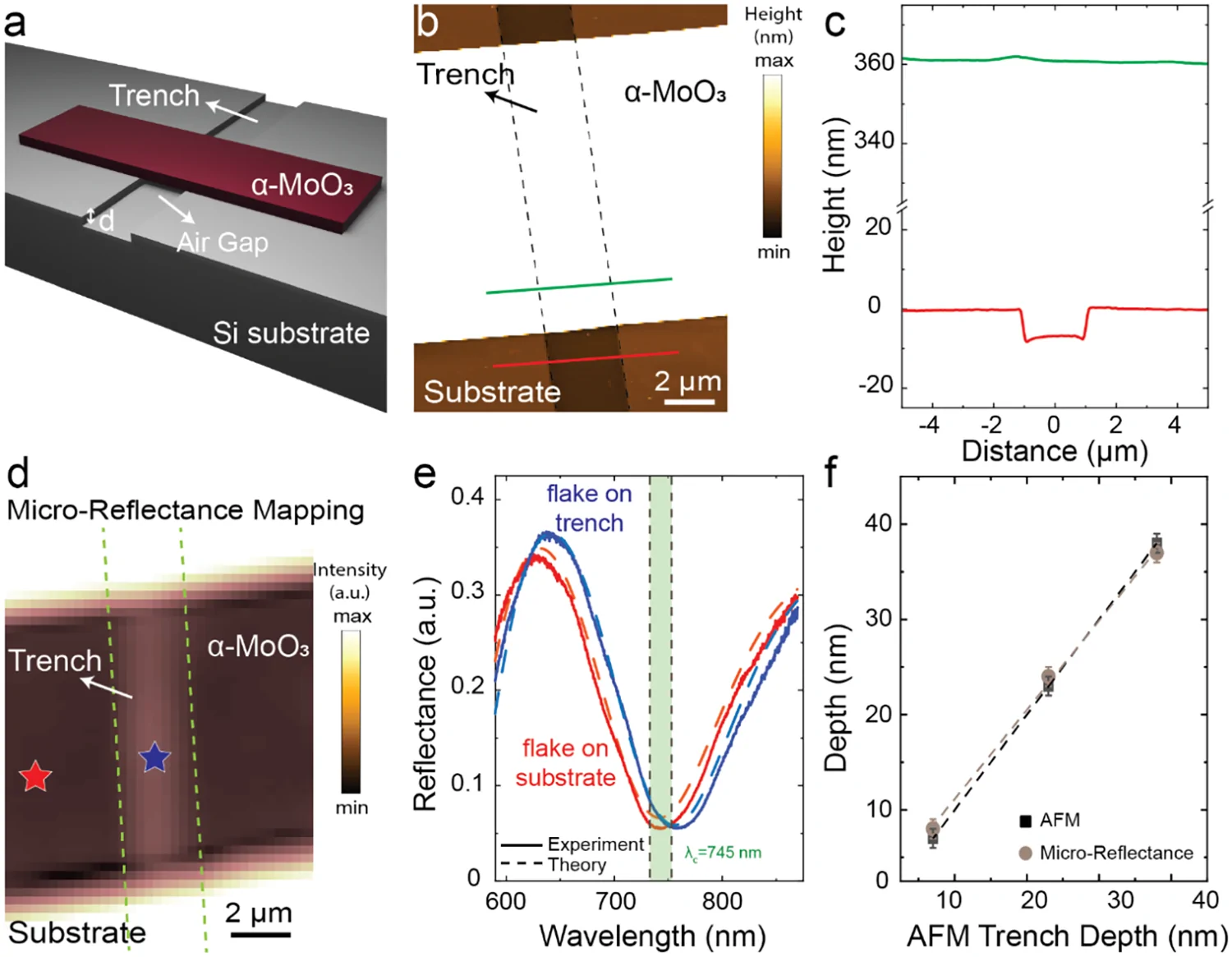
Detection and quantification of buried air gaps via microreflectance mapping. (a) Schematic illustration of the calibration samples, consisting of α-MoO_3_ flakes transferred over rectangular trenches of varying depths *d* fabricated on Si substrates. (b) AFM topography map of a 360 nm-thick α-MoO_3_ flake suspended over a 7 nm deep trench. The featureless flake surface indicates that the flake does not fold down into the cavity. (c) AFM height profiles along the red and green lines in (b). (d) Microreflectance map obtained with a step size of 500 nm on the same region shown in (b). The image contrast is obtained by integrating the reflectance signal around the spectral minimum within a ± 10 nm spectral window centered at λ_C_ = 745 nm for each pixel, which is marked with a shadowed green rectangle in (e). Unlike the AFM topography, the optical map clearly resolves the underlying trench due to a shift of the reflectance spectral minimum caused by the air gap between the flake and the substrate. (e) Experimental optical reflectance spectra (solid lines) extracted from the supported region of the flake (on Si substrate, red star in (d)) and from the trench region (on air gap, blue star in (d)). Fitting the data to the TMM calculations (dashed lines) yields an extracted air gap height of 8 nm, consistent with the nominal trench depth of 7 nm. (f) Correlation plot comparing the optically extracted air gap depths (circular gray dots) against the AFM-measured trench depths (squared black dots) for three samples.

As a prerequisite for the quantitative assessment of interface inhomogeneities in vdW stacks, we first establish the optical constants of the constituent materials. We focus on h-BN and α-MoO_3_, two polar materials widely used in polaritonics. In addition, h-BN is of particular strategic importance due to its widespread use as an encapsulant for preserving the electronic quality of graphene^28,40^ or TMDs.^41,42^

To extract the refractive indices, we performed microreflectance measurements on single, micromechanically exfoliated h-BN and α-MoO_3_ flakes transferred onto Si substrates, whose optical constant is well-known.^43^ The experimental spectra were fitted using the TMM. This model relates the reflectance of any stratified medium to the refractive index, *n*(*λ*), and thickness, *t*, of each layer, i.e., *R* = *R*(*λ*, *n*_*i*_(*λ*), *t*_*i*_),*i* = 0,···,*N* + 1, considering *N* to be the number of layers excluding substrate (Si) and superstrate (air) (see Supporting Information, Note S1). The model also accounts for the numerical aperture (NA) of the microscope objective by integrating the reflectance over the range of incidence angles determined by the NA^44^ (see Supporting Information, Note S2). The thicknesses of the h-BN and α-MoO_3_ flakes used in the model range from 10 to more than 400 nm and were independently determined by AFM measurements.

Since h-BN and α-MoO_3_ are transparent in the visible range, we modeled their refractive indices using the Cauchy dispersion relation, assuming negligible absorption (*k* ≈ 0)

1 $$n\left(\lambda \right)=A+\frac{B}{\lambda^{2}}$$

We first extracted the refractive index of h-BN by simultaneously fitting the reflectance spectra of several h-BN flakes with different thicknesses to this model (Figure 1b,c). The experimental reflectance was calculated as the ratio between the reflected and incident light intensities. The incident spectrum was estimated by measuring the reflected intensity from the reference Si substrate and normalizing it by the theoretical TMM Si reflectance (see Supporting Information, Note S3). In the global fitting procedure, the Cauchy parameters *A* and *B* were shared by all flakes, while the AFM-measured thickness of each individual flake was allowed to vary within ±3 nm. This range accounts for the AFM thickness uncertainty due to the nonflat surface morphology observed in most flakes. The same procedure was then applied to several α-MoO_3_ flakes. The extracted fitting parameters are *A*_*h*–BN_ = 2.412 ± 0.002 and *B*_*h*–BN_ = (2.42 ± 0.04)·10^–2^ μm^2^ for h-BN, and *A*_α–MoO_3__ = 2.523 ± 0.002 and *B*_α–MoO_3__ = (4.98 ± 0.05)·10^–2^ μm^2^ for α-MoO_3_. The experimentally obtained refractive indices for both materials are presented in Figure 1d. The corresponding goodness-of-fit metrics and additional details of the fitting procedure are provided in Table S1 and Supporting Information, Note S4. A sensitivity analysis of the refractive index fitting procedure, including the influence of the initial fitting parameters, AFM thickness uncertainty, smoothing conditions, wavelength range, and calibration-flake selection, is provided in Supporting Information, Note S5.

We note that the extracted refractive index values are approximately 10% higher than those previously reported,^45,46^ likely due to differences in crystal quality or exfoliation yield, highlighting the importance of in situ calibration for precise optical modeling. In addition, whereas h-BN is in-plane isotropic, α-MoO_3_ is in-plane anisotropic material. Since our approach does not account for the polarization of the incident or reflected light, the extracted refractive index should be regarded as an effective value, as the present configuration is not sensitive to the crystallographic orientation of the flakes. Nevertheless, the agreement between the experimental spectra and the model remains very good. A sensitivity analysis of the effective-index approximation for biaxial α-MoO_3_ is provided in Supporting Information, Note S9.

Furthermore, to enable in situ characterization of flakes exfoliated on PDMS transfer stamps, we experimentally determined the refractive index of the commercial PDMS stamps used in this work. In this work, we use commercial Gel-Pak WF films composed of a PDMS-based gel layer bonded to a polyester backing, hereafter referred to as PDMS-polyester transfer stamps (see Experimental Section). To this end, we followed a procedure like that described above, replacing the substrate in the optical model with the PDMS-Polyester stamp as the unknown optical medium while keeping the remaining parameters fixed, namely the flake thickness and its refractive index. The flake thicknesses were determined independently by AFM after transferring the same flakes onto Si substrates. This procedure yields an average refractive index of *n*_PDMS–Polyester_ = 2.14 ± 0.08, which remains rather constant over the investigated wavelength range from 500 to 800 nm. Knowing this value enables the direct determination of vdW flake thicknesses prior to assembly, as illustrated in Figure 1e,f, where reflectance measurements on two regions of an α-MoO_3_ flake on PDMS-Polyester stamps are used to extract the corresponding thicknesses using the TMM formalism.

To validate the experimentally extracted indices of h-BN and α-MoO_3_, we performed a blind test on a separate set of flakes. In particular, microreflectance measurements were carried out on several h-BN and α-MoO_3_ flakes transferred on Si substrates. By fixing *n*(λ) to the calibrated values obtained, the flake thicknesses were determined solely through TMM fitting of the reflectance spectra (see Supporting Information, Note S6). The extracted values for both α-MoO_3_ and h-BN flakes were subsequently compared with AFM measurements. Figure 1g shows this comparison, revealing excellent agreement, with systematic errors of 4% for h-BN and 1% for α-MoO_3_, calculated as the deviation of the fitted slope from unity. As mentioned above, α-MoO_3_ is an optically biaxial crystal and therefore, the extracted value should be regarded as an effective refractive index. Note that, in line with previous works, this approximation allows us to employ the computationally simpler isotropic TMM instead of the full anisotropic formalism.^37,39^ Moreover, and more importantly for practical applications in the fabrication of polaritonic heterostructures, we have demonstrated that it is possible to determine the thickness of single vdW slabs directly on the PDMS-Polyester stamp, eliminating the need for intermediate transfer steps. We have implemented these calibrations into an open-source software tool designed for the instant, in situ determination of flake thickness (see Associated Content).

Furthermore, we show that this procedure can be extended to hyperspectral mapping over large areas, both for flakes transferred onto substrates and for fully assembled vdW heterostructures, providing spatially resolved maps with a lateral resolution of approximately 1 μm, in which nanometric air gaps and other interfacial inhomogeneities between constituent layers can be revealed. Details of the experimental setup used to acquire the microreflectance maps are provided in the Experimental Section. To demonstrate this capability, we first fabricated calibration samples consisting of α-MoO_3_ flakes transferred onto Si substrates with prepatterned trenches of varying depths (7, 23, and 38 nm), as measured by AFM (Figure 2a,b). This engineered structure creates well-defined nanometric air gaps beneath the flakes, enabling us to assess the ability of microreflectance mapping to resolve such buried features. The AFM characterization confirmed that the flakes remained fully suspended over the trenches without folding down into the air gaps (Figure 2c). Importantly, while the AFM topography of the α-MoO_3_/7 nm-trench sample shows a featureless and flat surface on the suspended region, rendering the underlying nanometer sized air gap effectively invisible (Figure 2b), the corresponding microreflectance map clearly resolves the buried air gap due to the underlying trench (Figure 2d). In the reflectance spectra, this translates into systematic shifts of the interference maxima and minima between the regions of the flake on the Si substrate and trench structure, directly indicating the presence of the air gap (Figure 2e).

Beyond qualitative detection, fitting the experimental reflectance spectra using the TMM enables the simultaneous extraction of both the flake thickness and the depth of the underlying air gap given by the trench. The thickness of the α-MoO_3_ flake is extracted from the reflectance measured on the region outside the trench; therefore, the air-gap thickness is the only free parameter in the fitting procedure. Figure 2f compares the trench depths measured by AFM with the values extracted from optical reflectance fitting, showing excellent agreement with absolute errors of around ∼1 nm and demonstrating the high sensitivity of the method to resolve buried nanometric features that are not accessible to conventional scanning probe techniques.

Subsequently, we demonstrate the capability of microreflectance mapping to identify and quantify interfacial inhomogeneities intrinsic to vdW assembly, such as nanobubbles and trapped air gaps. We first focus on the detection and analysis of nanobubbles in an α-MoO_3_ flake transferred onto a Si substrate. The AFM image in Figure 3a shows a nanobubble formed after transfer, approximately 10 nm in height and 5 μm in diameter. Remarkably, this nanobubble can be resolved in the microreflectance map through a change in the optical reflectance contrast, as indicated by the red dashed circle in Figure 3b. Its appearance across multiple pixels in the map demonstrates the method’s high spatial resolution of approximately 1 μm. The TMM fits to the experimental optical reflectance spectra obtained on the substrate-supported and nanobubble regions (dashed lines in Figure 3c) yield a flake thickness of *t*_1_^TMM^ = 217 nm (RMSE = 0.011) and a bubble height of *h*_bubble_^TMM^ = 9 nm (RMSE = 0.02), in very good agreement with the AFM measurements (*t*_1_^AFM^ = 220 nm and *h*_bubble_^AFM^ = 10 nm), which confirms the reliability of microreflectance spectroscopy combined with TMM analysis for quantitatively resolving nanometric air gaps in vdW heterostructures.

**Figure 3:**
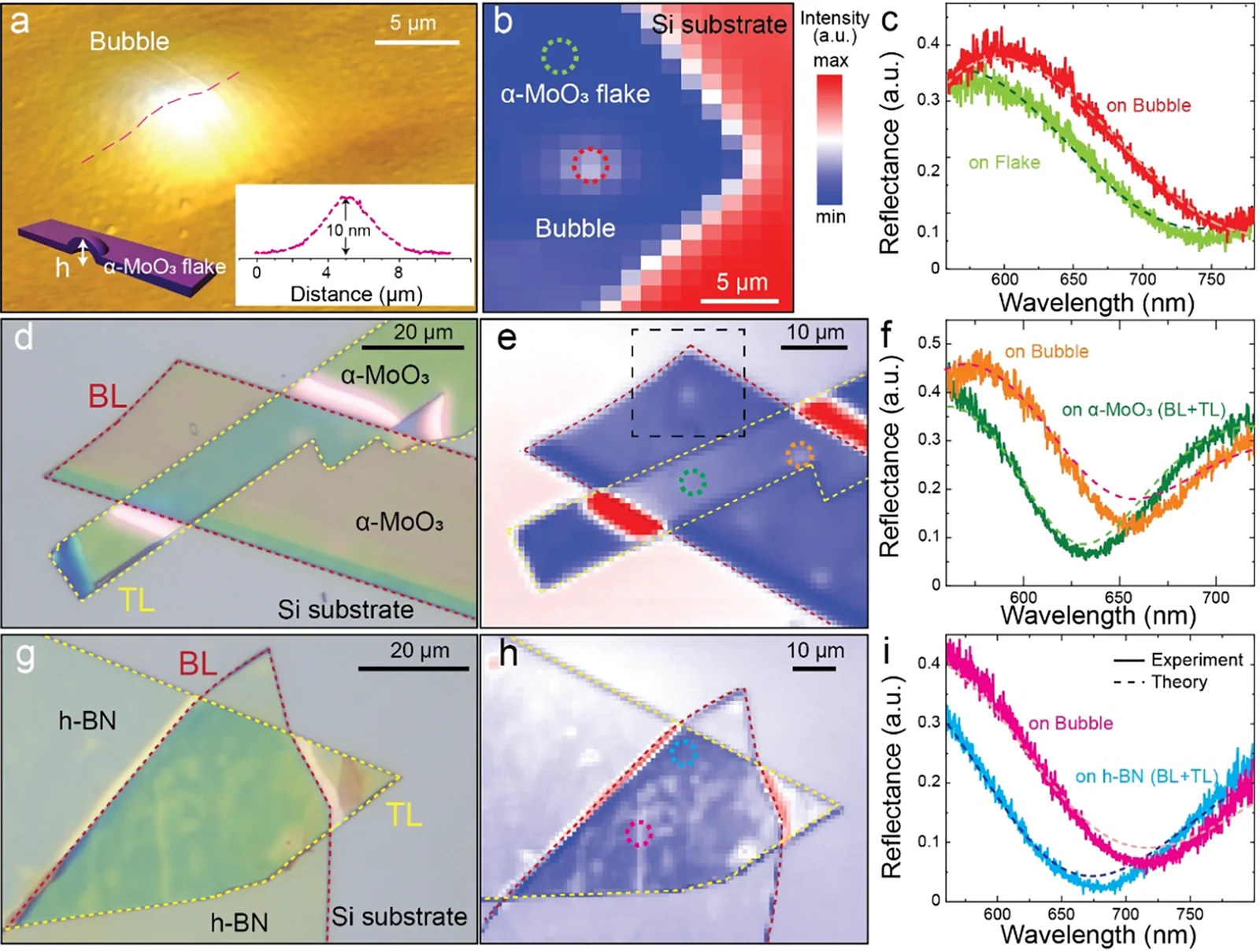
Quantitative imaging of nanobubbles at vdW twisted stacks interfaces. (a) AFM topography of an α-MoO_3_ flake transferred onto a Si substrate, revealing a nanobubble with a height of ∼10 nm. The inset sketch illustrates the geometry of a nanobubble with height *h* (left) and the AFM profile over the nanobubble along the dashed red line (right). (b) Microreflectance map of the α-MoO_3_ flake corresponding to the region marked by the dashed black rectangle in (e), revealing the nanobubble highlighted by the red dashed circle and shown in (a). (c) Experimental reflectance spectra, shown with solid lines, acquired at the flat region (green dashed circle in (b)) and at the center of the nanobubble, together with the corresponding theoretical TMM fits (dashed lines). (d) Optical image and (e) corresponding microreflectance map of an α-MoO_3_ heterostructure composed of a top 200 nm-thick layer (TL) stacked onto a bottom 220 nm-thick layer (BL) on a Si substrate. The color contrast observed in the optical images arises from thin-film interference under white-light illumination in the optical microscope and reflects local thickness variations across the stacked flakes. The microreflectance map reveals a distinct optical contrast associated with interfacial imperfections between the two flakes forming the structure. (f) Reflectance spectra acquired at the flat region of the structure (green dashed circle in (e)) and at the center of a nanobubble (orange dashed circle in (e)), together with the corresponding theoretical TMM fits (dashed lines). (g) Optical image and (h) corresponding microreflectance map of an h-BN heterostructure composed of a top 100 nm-thick layer (TL) stacked onto a bottom 120 nm-thick layer (BL) on a Si substrate. (i) Reflectance spectra acquired at a flat region of the structure (blue dashed circle in (h)) and at the center of a nanobubble (pink dashed circle in (h)), together with the corresponding theoretical TMM fits (dashed lines). The step size for the microreflectance maps was set to 1 μm.

We extend the analysis to the interfaces formed between stacked flakes in heterostructures based on vdW materials, where interfacial gaps and thickness nonuniformities may arise during the assembly process. In particular, we investigate α-MoO_3_ and h-BN homostructures, two material platforms widely used in polaritonics, for which the quality and uniformity of the interfacial contact are crucial. Figure 3d,g show the optical image of α-MoO_3_ and h-BN bilayer heterostructures on a Si substrate, respectively. The corresponding microreflectance maps (Figure 3e,h) enable rapid visualization of air gaps present in the bottom layer and at the structure interface, where the presence of such nonuniformities is revealed by a shift in the optical reflectance spectra with respect to the surrounding regions, as shown in Figure 3f,i. In particular, the interfacial quality in the structures is quantified by fitting TMM calculations to the experimental spectra acquired on top of the α-MoO_3_ and h-BN structures and at bubble regions as shown in Figure 3f,i, respectively. In particular, whereas a uniform interface is found in the overlap region of the α-MoO_3_ homostructure (green dashed circle in Figure 3e) together with a bubble (orange dashed circle in Figure 3e) with height *h*_bubble_^TMM^ = 18 nm (RMSE = 0.07; *h*_bubble_^AFM^ = 20 nm), the h-BN heterostructure exhibits noncontact regions with interfacial air gaps of ∼ 12 nm (RMSE = 0.03; pink dashed circle in Figure 3h) together with areas showing a closer contact between the stacked flakes with no measurable air gap (blue dashed circle in Figure 3h). This behavior is likely related to the lower mechanical rigidity of h-BN flakes compared with α-MoO_3_, as well as to their smaller thickness, which makes them more susceptible to local deformation during the stacking process. At this point, we emphasize the high sensitivity of the measurements to resolve and quantify interfacial nonuniformities that remain imperceptible in conventional optical or AFM images.

Finally, to validate the accuracy of our microreflectance methodology in quantifying buried interfacial features and to assess the influence of nanometric air gaps on the optical response of propagating PhPs in polaritonic slabs, we employed s-SNOM to visualize the propagation of PhPs on α-MoO_3_ flakes transferred on *d* = 7 and 23 nm deep trenches (Figure 4a). Since the momentum and confinement of PhPs in anisotropic slabs are critically dependent on the dielectric permittivity of the substrate,^12,47^ the presence of an air gap is expected to induce a measurable modification in the polaritonic dispersion. This high sensitivity to the dielectric environment arises from the surface-like nature of PhPs in biaxial crystals, such as α-MoO_3_, where the associated electromagnetic fields are tightly compressed within the slab and its immediate vicinity (λ*_p_* ≪ λ_0_), where λ_*p*_ and λ_0_ are the wavelength of PhPs and incident radiation, respectively. Consequently, PhPs act as an ultrasensitive nanoscopic probe, capable of detecting subtle variations in the substrate topography such as a nanometric step from Si to air regions. Indeed, near-field amplitude (see Experimental Section) images recorded at ω = 880 cm^–1^ for trenches with a depth of *d* = 7 nm (Figure 4b) reveal a distinct phase shift in the PhPs interference fringes as the wavefront propagates from the Si-supported region to the area suspended over the trench. Profiles extracted along the propagation direction (Figure 4c) quantify this effect, showing a noticeable variation in the polariton wavelength, λ_*p*_, induced by the change in the dielectric environment due to the underlying air gap. In particular, λ_*p*_ increases by 6% in the air-gap region for *d* = 7 nm at ω = 880 cm^–1^. By comparing the experimental dispersion relation extracted from the suspended region against analytical calculations^48^ (Figure 4d), we find that the observed momentum shift corresponds precisely to the nominal trench depth of 7 nm (see Supporting Information, Notes S6 and S7). Similar observations are obtained for trenches with a depth of *d* = 23 nm (Figure 4e–g). While s-SNOM is a powerful tool for visualizing polariton propagation in polaritonic slabs and resolving trapped air gaps in heterostructures, it is inherently a slow, local scanning technique. The excellent agreement demonstrated here between the nanoscopic near-field dispersion and our far-field results not only validates the accuracy of the extracted gap depths but also establishes microreflectance mapping as a powerful, high-throughput alternative for the stringent quality control of interfaces required for an optimal assembly of vdW heterostructures in polaritonics, and particularly in twisted photonic structures or twistoptics. We note that here, although α-MoO_3_ serves as a representative polaritonic platform for the s-SNOM studies, the conclusions are expected to extend to other polaritonic materials and heterostructures.

**Figure 4:**
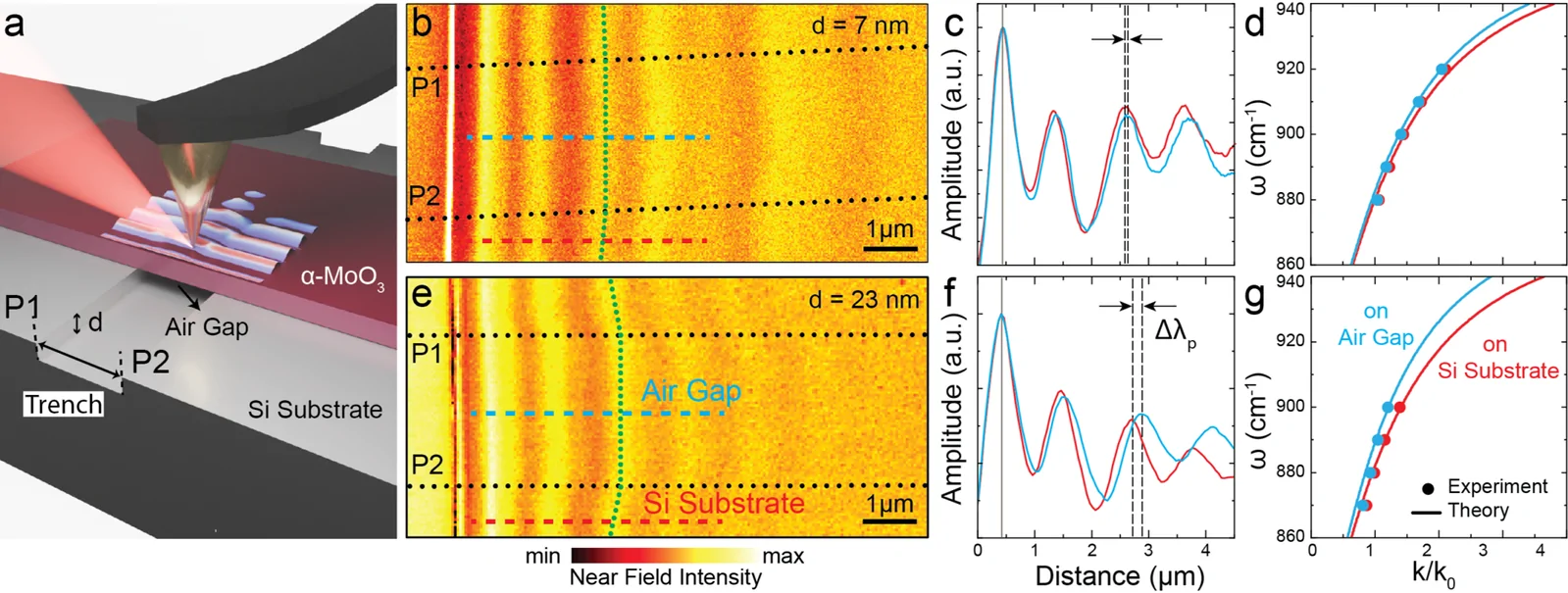
Extraction of trench depth via near-field nanoimaging of PhPs. (a) Schematic illustration of the optical scheme used in s-SNOM to probe PhPs in a 365 nm-thick α-MoO_3_ flake suspended over a Si trench of depth *d*. (b) Near-field amplitude image (*s*_3_) recorded at ω = 880 cm^–1^ for a trench depth of *d* = 7 nm. Black dotted lines delineate the trench boundaries, while green dotted lines highlight the propagating polaritonic wavefronts. (c) Normalized amplitude profiles extracted along the propagation direction over the Si substrate (red dashed line in (b)) and over the trench (blue dashed line in (b)). Vertical solid lines indicate the fringe maxima, highlighting the phase shift and the modification of PhPs wavelength marked with vertical dashed lines (Δλ_*p*_), induced by the underlying air gap. (d) Experimental dispersion data extracted for the substrate-supported (red dots) and suspended (blue dots) regions, overlaid with the corresponding analytical dispersion curves (solid lines). (e–g) Analogous measurements and analysis for a trench depth of *d* = 23 nm. Comparing (c, f) demonstrates a clear depth-dependent variation in λ_*p*_, confirming the sensitivity of PhPs to the vertical dimension of the buried interface, i.e., presence of air gaps with varying heights.

## 4 Conclusions

In this work, we have established microreflectance mapping as a sensitive, robust, and nondestructive technique for the characterization of the interface quality in vdW stacks. By coupling high-resolution hyperspectral imaging with a TMM-based fitting algorithm, we have addressed critical bottlenecks in the fabrication of devices relevant to polaritonics: the difficulty of determining layer thickness directly on widely used soft viscoelastic PDMS stamps and the inability of standard topographic probes to detect buried interfacial inhomogeneities in vdW heterostructures. First, by extracting the optical constants of representative polar materials supporting PhPs (h-BN and α-MoO_3_) and commercial PDMS-Polyester films, we enabled the in situ determination of flake thickness directly on transfer PDMS-Polyester stamps with systematic errors below 4%. This capability significantly streamlines the fabrication workflow by eliminating the risk and time associated with intermediate transfer steps previously required for AFM characterization. Second, and most crucially, we demonstrated the technique’s unique ability to resolve buried interface inhomogeneities that are invisible to topographic inspection. We successfully detected and quantified air gaps as thin as 7 nm in calibration structures and resolved nonuniformities on the adhesion between individual flakes in stacked heterostructures. As validated by our near-field s-SNOM measurements, detecting these nanoscopic inhomogeneities is key for polaritonics, where the propagation of PhPs is strictly dependent on the dielectric continuity of the interface. Finally, by implementing this methodology into an open-source software tool, we provide the 2D materials community with an accessible solution that can be readily integrated into conventional optical microscopy setups for both rapid thickness identification and stringent quality control of interfaces. The microreflectance mapping presented here can also be extended to other vdW materials and heterostructures, including those based on monolayer TMDs, where variations in the local dielectric environment due to possible presence of air gaps or contaminants at the interface with encapsulating h-BN or other vdW materials can significantly modify the optical response through dielectric screening effects.^49^ This work lays the foundation for the reproducible assembly of high-quality heterostructures, a prerequisite for unlocking the full potential of polaritonics in twisted slabs (twistoptics) and next-generation nanophotonic devices.

## Supplementary Material



## Data Availability

The code used for the analysis and simulations in this work is publicly available at GitHub: https://github.com/juliagprieto/Reflectra

## Notes

TMM model and numerical-aperture treatment; experimental reflectance calculation; h-BN and α-MoO_3_ refractive index extraction and sensitivity analysis; flake-thickness determination; microreflectance layer sensitivity analysis; simulated air gap detection sensitivity versus air gap and overlayer thickness; effective-index approximation for biaxial α-MoO_3_; polariton-wavelength sensitivity to nanometric air gaps; dispersion relation of highly confined PhPs in thin-film bilayers; dielectric function of α-MoO_3_ (PDF)
