# Supplementary material for: Unveiling Buried Air Gaps and Interface Inhomogeneities in van der Waals Stacks via Microreflectance Mapping for Twistoptics Applications
Source: ACS Appl Nano Mater. 2026 Aug 18;9(34):16286–96. doi: 10.1021/acsanm.6c02110 (PMC13532039; doi:10.1021/acsanm.6c02110)
Supplement: Supplementary file 1 [file an6c02110_si_001.pdf]

Supporting Information

Unveiling Buried Air Gaps and Interface Inhomogeneities in van der Waals Stacks via Micro-Reflectance Mapping for Twistoptics Applications

Julia G. Prieto[1,2*], Adam Roselló[1,3], Abel Martínez-Suárez[1,2], Enrique Terán-García[1,2], Christian Lanza[1,2], Yolanda Álvarez[1], Stephan Kasemann[4], Stefan Partel[4], Kenji Watanabe[5], Takashi Taniguchi[6], Adrián Fernández-Gavela[1], Dimas G. de Oteyza[2,3], Luis Javier Martínez[2], Aitana Tarazaga Martín-Luengo[1,2], Pablo Alonso-González[1,2*], Javier Martín-Sánchez [1,2*]

[1]*Department of Physics, University of Oviedo, Oviedo 33006, Spain.*
[2]*Nanomaterials and Nanotechnology Research Center (CINN), CSIC-University of Oviedo-Principality of Asturias, Avenida de La Vega 4-6 El Entrego, San Martin Del Rey Aurelio, 33940 Asturias, Spain.*
[3]*Donostia International Physics Center, 20018 San Sebastian, Spain.*
[4]*Research Centre for Microtechnology, FHV – Vorarlberg University of Applied Sciences, Campus V, Hochschulstraße 1, 6850 Dornbirn, Austria.*
[5]*Research Center for Electronic and Optical Materials, National Institute for Materials Science, 1-1 Namiki, Tsukuba 305-0044, Japan*
[6]*Research Center for Materials Nanoarchitectonics, National Institute for Materials Science, 1-1 Namiki, Tsukuba 305-0044, Japan*

*Corresponding authors: juliagprieto@uniovi.es; pabloalonso@uniovi.es; javiermartin@uniovi.es

The Supporting Information of this work consists of twelve notes displaying complementary results and calculations to those described in the main manuscript and is structured as follows:

- **Note S1.** Transfer matrix method.
- **Note S2.** Consideration of the numerical aperture of the objective.
- **Note S3.** Calculation of the experimental reflectance.
- **Note S4.** Extraction of refractive indices of h-BN and α-MoO_3 flakes.
- **Note S5.** Sensitivity analysis of the refractive-index fitting procedure.
- **Note S6.** Determination of flake thicknesses.
- **Note S7.** Visibility and accuracy on the estimation of large nanoscale air gaps.
- **Note S8.** Simulated air-gap detection sensitivity as a function of air-gap thickness and overlayer thickness.
- **Note S9**. Sensitivity analysis of the effective-index approximation for biaxial α-MoO_3.
- **Note S10**. Polariton-wavelength sensitivity to nanometric air gaps in α-MoO_3.
- **Note S11**. Derivation of the dispersion relation of highly confined phonon-polaritons (PhPs) in bilayers of thin film materials.
- **Note S12**. Dielectric function of α-MoO_3.

Note S1. Transfer matrix method.

The transfer matrix method (TMM) model is a matrix formalism describing the propagation of light in a stratified medium[1]. The first and last layers are considered semi-infinite. The materials studied in this work, h-BN and α-MoO_3, are treated within an effective isotropic, nonmagnetic approximation $\mu \approx \mu_0$.

At each interface ij of a stratified medium, one part of the beam is reflected into the first layer, i, while the other is transmitted to the next layer, j. Therefore, the electric field within each layer i can be expressed as a sum of incident ($\vec{E}_i^+$) and reflected ($\vec{E}_i^-$) fields:

$$\vec{E}_i = \vec{E}_i^+ + \vec{E}_i^- . \qquad (S1)$$

Besides, waves travelling through a layer j of thickness t_j experience a phase delay given by $k_j cos\theta_j t_j$, $k_j = \frac{2\pi}{\lambda}\hat{n}_j$ being the wavenumber, meaning that

$$E_i^{\pm} = E_j^{\pm} e^{\mp i k_i cos\theta_i d_i}. \qquad (S2)$$

For *s* polarization waves, the magnetic field $\vec{H}$ is in the plane of incidence, and the continuity of the electric and magnetic fields at the interface ij leads to the system of equations:

$$E_i^+ + E_i^- = E_j^+ e^{-ik_i cos\theta_i d_i} + E_j^- e^{+ik_i cos\theta_i d_i}, \qquad (S3)$$

$$\left(E_i^+ - E_i^-\right)\hat{n}_i \cos(\theta_i) = (E_j^+ e^{-ik_i cos\theta_i d_i} - E_j^- e^{+ik_i cos\theta_i d_i})\hat{n}_j \cos\left(\theta_j\right). \qquad (S4)$$

This system can be written in matrix form as:

$$\begin{pmatrix} E_i^+ \\ E_i^- \end{pmatrix} = P_i D_{ij} \begin{pmatrix} E_j^+ \\ E_j^- \end{pmatrix} \qquad (S5)$$

Where P_i is a matrix describing the phase change of the wave as it propagates through the layer i:

$$P_i = \begin{pmatrix} e^{-ik_i cos\theta_i d_i} & 0 \\ 0 & e^{+ik_i cos\theta_i d_i} \end{pmatrix}, \qquad (S6)$$

while the matrix D_{ij} describes the phase and amplitude variation of the wave at the interface ij, and can be written as a function of the reflection r_{ij} and transmission t_{ij} coefficients in the following way:

$$D_{ij} = \frac{1}{t_{ij}} \begin{pmatrix} 1 & r_{ij} \\ r_{ij} & 1 \end{pmatrix}. \qquad (S7)$$

On the other hand, the reflection and transmission coefficients are

$$r_{ij}^S = \frac{\hat{n}_i \cos(\theta_i) - \hat{n}_j \cos\left(\theta_j\right)}{\hat{n}_i \cos(\theta_i) + \hat{n}_j \cos\left(\theta_j\right)}, \; t_{ij}^S = \frac{2\hat{n}_i \cos(\theta_i)}{\hat{n}_i \cos(\theta_i) + \hat{n}_j \cos\left(\theta_j\right)}. \qquad (S8)$$

In the case of p polarization, the continuity conditions lead to an analogous matrix form but with parameters:

$$r_{ij}^{P} = \frac{\hat{n}_j \cos(\theta_i) - \hat{n}_i \cos(\theta_j)}{\hat{n}_j \cos(\theta_i) + \hat{n}_i \cos(\theta_j)},\ t_{ij}^{P} = \frac{2\hat{n}_i \cos(\theta_i)}{\hat{n}_j \cos(\theta_i) + \hat{n}_i \cos(\theta_j)}. \qquad (S9)$$

Repeating the same argument iteratively, it can be demonstrated that for a multilayer system of N layers of refractive indices $n_1,..., n_N$, in which the superstrate (n_0) and substrate (n_{N+1}) are semi-infinite media, Eq. S7 can be recast as follows:

$$\begin{pmatrix} E_0^+ \\ E_0^- \end{pmatrix} = D_{01} \cdot P_1 \cdot D_{12} \cdot \ldots \cdot P_N \cdot D_{N,N+1} \begin{pmatrix} E_{N+1}^+ \\ E_{N+1}^- \end{pmatrix} = M \begin{pmatrix} E_{N+1}^+ \\ E_{N+1}^- \end{pmatrix}. \qquad (S10)$$

Considering that $E_{N+1}^- = 0$, the reflection coefficient depends on the matrix elements of M in the following form:

$$r = \frac{E_0^-}{E_0^+} = \frac{M_{21}}{M_{11}}. \qquad (S11)$$

In the case of a single layer, it can be shown that

$$r^{s,p} = \frac{r_{01}^{s,p} e^{-ik_1 \cos(\theta_1) t_1} + r_{12}^{s,p} e^{ik_1 \cos(\theta_1) t_1}}{r_{01}^{s,p} r_{12}^{s,p} e^{ik_1 \cos(\theta_1) t_1} + e^{-ik_1 \cos(\theta_1) t_1}}, \qquad (S12)$$

where the subscripts 0, 1, 2 are assigned as air, single layer and substrate, respectively. The reflectance is then given by $R^{s,p} = |r^{s,p}|^2$.

Note S2. Consideration of the numerical aperture of the objective.

Following Refs.[2–4], we do not assume normal incidence but a distribution of angles of incidence due to the numerical aperture (NA) of the involved optical elements[4]. Besides, the incident light is considered unpolarized; therefore, the final reflectance is obtained by averaging the s- and p-polarized reflectances. The reflectance is then given by[4]:

$$R(\lambda) = \frac{1}{2} \sum_{m=1}^{M} w_m (R^s(\lambda, \theta_m) + R^p(\lambda, \theta_m)), \qquad (S13)$$

where $M$is the number of discrete angles into which we divide the maximum incidence angle, $\theta_m = \frac{m}{N-1}\arcsin(NA)$, and w_m is a weight function reflecting the distribution of angles of the reflectance:

$$w_m = \frac{2\text{m} - 1}{M^2}. \qquad (S14)$$

In the first experimental configuration, namely the modified optical microscope, spatial resolution is determined by a multimode optical fibre placed directly after the objective, which angularly restricts the cone of collected rays. Consequently, the effective numerical aperture of the detection system is limited by the minimum between the numerical aperture of the objective and that of the fibre; in this case, $NA_{\text{fiber}} = 0.22$.

In contrast, in the micro-reflectance mapping setup the pinhole acts purely as a spatial selector, because it is placed in an image plane. Therefore, the relevant numerical aperture is that of the objective itself, $NA = 0.7$.

However, the effect of the numerical aperture becomes significant only for values above approximately 0.5. This is illustrated in **Figure S1**, where the theoretical reflectance of a 200 nm-thick α-MoO_3 flake on a silicon substrate is calculated for several numerical apertures. We extended this analysis to flake thicknesses ranging from 10 to 400 nm and found that assuming normal incidence introduces an error in extracted thickness of less than 2% for experimental setups with NA below 0.7. Consequently, refractive index extraction and in-situ thickness estimation in the modified microscope can be reliably performed using the simpler normal-incidence approximation, which also reduces computation time.

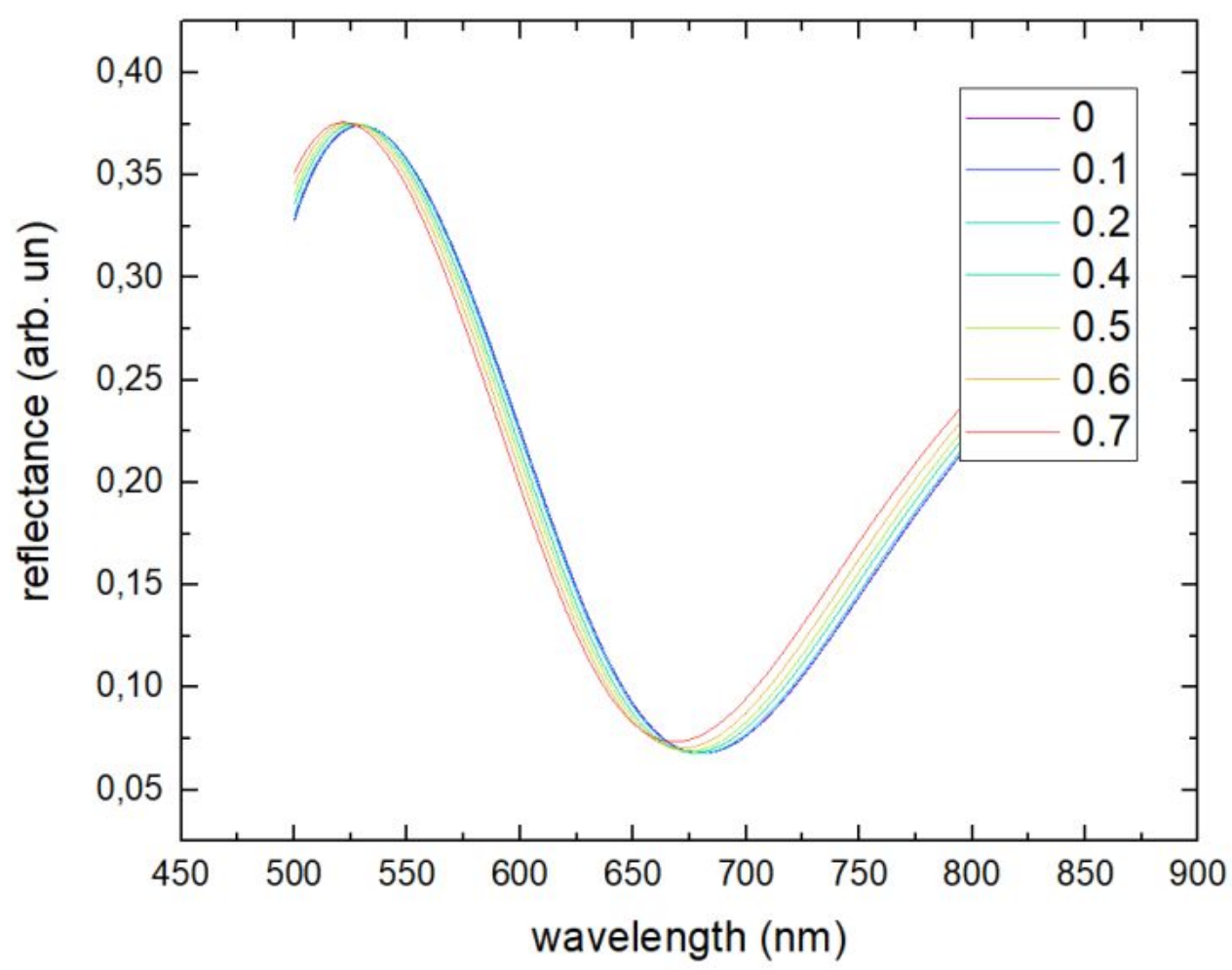

Figure S1. Theoretical reflectance of a 200 nm thick α-MoO_3 flake on a silicon substrate for several numerical apertures**.**

Note S3. Calculation of the experimental reflectance.

Experimentally, the reflectance is defined as the ratio between the reflected and incident light intensities:

$$R(\lambda) = \frac{I_R(\lambda)}{I_0(\lambda)}. \tag{S15}$$

Since the incident light intensity $I_0(\lambda)$ cannot be directly measured in our optical configuration, a bare reference substrate was used to estimate the effective incident spectrum. The effective incident spectrum was then estimated as

$$I_0(\lambda) = \frac{I_{R,S}(\lambda)}{R_S^{TMM}(\lambda)}, \tag{S16}$$

where $I_{R,S}(\lambda)$ is the reflected intensity at the substrate, and $R_S^{TMM}(\lambda)$ is the theoretical reflectance of the substrate. In this work, Si substrates were used as reference substrates, and the Si optical constants were extracted from Ref[5]. The refractive index of air was taken as $n_{air} \simeq 1$. Combining Eqs. (S15) and (S16), the experimental reflectance of a flake was calculated as:

$$R_{exp}(\lambda) = \frac{I_{R,flake}(\lambda)}{I_{R,S}(\lambda)} R_S^{TMM}(\lambda). \tag{S17}$$

Thus, the fitting routine does not operate on raw-detector intensities, but on the experimental reflectance spectra. This normalization removes the wavelength-dependent response of the illumination source, optical path, and detector, assuming that these contributions are common to the flake and the substrate measurements and that both spectra are acquired under the same optical alignment and acquisition conditions.

Note S4. Extraction of refractive indices of h-BN and α-MoO_3 flakes.

The refractive index of each material was described using a Cauchy dispersion relation,

$$n(\lambda) = A + \frac{B}{\lambda^2}, \tag{S18}$$

where λ is expressed in μm, so that B has units of μm^2. Absorption was neglected in the fitted spectral range, i.e. $k \approx 0$.

To extract the h-BN and α-MoO_3 refractive indices, the experimental reflectance spectra of several flakes with independently measured AFM thicknesses were fitted simultaneously to the theoretical reflectance calculated using the TMM. In the global calibration, the Cauchy parameters A and B were shared by all calibration flakes of a given material, while the thickness of each flake was treated as an independent fitting parameter constrained within the AFM uncertainty interval.

For each flake j, the residual was defined as

$$r_j(\lambda) = R_{exp,j}(\lambda) - R_{TMM,j}(\lambda, A, B, d_j), \tag{S19}$$

where $R_{exp,j}(\lambda)$ is the Whittaker-smoothed experimental reflectance[6], $R_{TMM,j}(\lambda, A, B, d_j)$ is the corresponding TMM reflectance and d_j is the thickness of the flake. The global fit was obtained by minimizing the sum of the squared residuals over all calibration flakes and wavelength points.

In practice, the smoothed experimental spectra from all calibration flakes were concatenated into a single one-dimensional array. The model function supplied to scipy.optimize.curve_fit returned the corresponding concatenated TMM spectra calculated using the shared A and B values and the individual fitted thicknesses d_j. Thus, the nonlinear least-squares routine minimized the difference between the concatenated experimental reflectance array and the concatenated TMM array.

Unless otherwise specified, the TMM calculations were performed at normal incidence. The effect of the finite numerical aperture of the objective is discussed separately in Note S3. For each fit, the extracted optical parameters were recorded together with several standard goodness-of-fit metrics, including the mean squared error (MSE), root mean squared error (RMSE), normalized RMSE (NRMSE), and maximum absolute residual. The MSE was calculated as the average of the squared residuals over all wavelength points and calibration flakes, providing a measure of the overall fitting error. The RMSE, defined as the square root of the MSE, has the same units as the reflectance. To facilitate comparison between different fitting conditions, the NRMSE was also evaluated by dividing the RMSE by the reflectance range of the corresponding dataset. In addition, the maximum absolute residual was monitored to identify localized wavelength regions where the disagreement between experiment and model was largest.

The final extracted Cauchy parameters and goodness-of-fit metrics are summarized in Table S1. The global fits yield RMSE values below 2% for both h-BN and α-MoO_3. These values confirm that the TMM reproduces the main spectral features of the calibration flakes spectra with good accuracy over the fitted wavelength range.

Material	A	σ_A	B (μm^2)	σ_B (μm^2)	MSE	RMSE	NRMSE (%)	Max. $\lvert r \rvert$
h-BN	2.412	0.002	0.0242	0.0004	$2.3 \cdot 10^{-4}$	$1.5 \cdot 10^{-2}$	4	0.04
α-MoO_3	2.523	0.002	0.0498	0.0005	$3.1 \cdot 10^{-4}$	$1.8 \cdot 10^{-2}$	5	0.05

Table S1. Cauchy parameters and goodness-of-fit metrics for h-BN and α-MoO_3 using a global refractive index calibration.

Note S5. Sensitivity analysis of the refractive-index fitting procedure.

To assess the influence of the fitting procedure described in Note S4, we performed a systematic sensitivity analysis of the refractive-index extraction. The analysis considered: (i) randomized initial conditions for the Cauchy parameters A and B; (ii) different AFM thickness bounds; (iii) different wavelength fitting windows; (iv) different Whittaker smoothing parameters, including comparison with the raw spectra; and (v) a leave-one-flake-out (LOFO) analysis, in which the global fit was repeated after removing one calibration flake at a time. Together, these tests evaluate the robustness of the fitted refractive index with respect to optimizer initialization, coupling to thickness uncertainty, spectral-window selection, preprocessing of the experimental spectra, and calibration-flake selection. This sensitivity-analysis was applied to the extraction of both the h-BN and α-MoO_3 refractive indices.

To assess whether the fitting routine converges to a stable solution rather than to an arbitrary local minimum, we performed a multi-start analysis using the normal-incidence TMM. In this test, the flake thicknesses were fixed to their AFM-measured values and only the Cauchy dispersion parameters A and B were optimized. For each run, the initial values of A and B were randomly sampled within broad intervals, $A \in [1, 5]$ and $B \in [0,1]$ μm^2, and the fit was repeated 500 times. For h-BN, the lowest-error basin was reached in 399/500 runs and yielded A = 2.409, B =0.0234 μm^2, with RMSE = 0.03. Two additional basins were identified, but both showed substantially larger errors, with approximately 19 and 23 times larger MSE values than that of the lowest-error basin. For α-MoO_3, the wide-range multi-start analysis showed even stronger convergence to the lowest-error solution. Out of 500 random initializations, 483 runs converged to the lowest-error basin, corresponding to 97% of all starts. This basin yielded $A = 2.542$ and $B = 0.0459\ \mu m^2$, with RMSE = 0.03. The second convergence basin showed a MSE approximately 24 times larger than that of the lowest-error basin. These results show that the fitted Cauchy parameters converge reproducibly to the same low-error basin over a wide range of initial conditions. Since the multi-start test was performed with fixed AFM thicknesses, it should be interpreted as an initialization-stability test for the optical parameters rather than as an exact repetition of the full bounded-thickness global calibration.

To evaluate whether the extracted Cauchy parameters depend on the assumed AFM thickness uncertainty, we repeated the full global calibration fit while varying the allowed thickness bounds around the AFM-measured values. In each fit, the same set of calibration flakes was used, the optical model was kept at normal incidence, and the fitted parameters were the shared Cauchy coefficients A and B, together with one thickness value per flake constrained to $d_{AFM} \pm \Delta d$. The tested bounds were $\Delta d = 1, 3$ and 5 nm, with $\Delta d = 3$ nm corresponding to the baseline calibration used in the main analysis. For h-BN, the fitted value of A remained highly stable over the tested range, with a relative spread of approximately 0.5% around the baseline value. The dispersion parameter B showed a larger dependence on the allowed thickness window, with a relative spread of approximately 10%. For α-MoO_3, the corresponding variations were 0.9% for A and 8% for B. In both materials, the NRMSE decreased when the allowed thickness interval was widened. However, the baseline value of $\Delta d = 3$ nm was retained because it more closely represents the experimental uncertainty associated with the AFM thickness measurements. These results indicate that the fitted offset parameter A is robust against the precise choice of AFM thickness window, whereas the dispersion parameter B exhibits a stronger coupling to the allowed thickness uncertainty.

Next, we evaluated whether the extracted Cauchy parameters A and B depend on the smoothing applied to the experimental reflectance before fitting. The calibration set was refitted using either the raw reflectance spectra or the Whittaker-smoothed reflectance spectra with $\lambda = 10^3, 10^4$ and 10^5. In all cases, each flake thickness was allowed to vary within $\pm$ 3 nm of its AFM-measured value. For h-BN, the extracted parameters remained essentially unchanged across all tested cases, with A changing by only about 0.002% and B by about 0.09%, while the NRMSE remained nearly constant around $4 \cdot 10^{-2}$. Similar results were obtained for α-MoO_3, for which A changed by only 0.006% and B by about 0.12%, with NRMSE values remaining around $5 \cdot 10^{-2}$. This confirms that the reported refractive-index dispersion is robust against the chosen Whittaker smoothing parameter.

To assess the dependence of the extracted Cauchy parameters on the spectral fitting range, we repeated the global calibration using different wavelength windows. In all cases, the same set of calibration flakes were used, and only the wavelength interval included in the fit was changed. Additional fits were performed by progressively removing the short-wavelength edge, the long-wavelength edge, or both edges of the spectrum. For each window, the fit optimized the shared Cauchy parameters A and B, together with one thickness value per flake constrained within ±3 nm of the AFM-measured thickness. All wavelength-window fits converged successfully. For h-BN, the largest deviation was $|\Delta n| = 1.05 \cdot 10^{-2}$ over the common range. For α-MoO_3, the corresponding value was $|\Delta n| = 8 \cdot 10^{-3}$. These results indicate that the extracted refractive index is not dominated by a specific spectral region within the fitted wavelength range.

Finally, to evaluate whether the extracted refractive index was dominated by any individual calibration spectrum, we performed a leave-one-out sensitivity analysis. Starting from the baseline global fit using all calibration flakes, the fit was repeated after removing one calibration flake at a time. In each LOFO fit, the shared Cauchy parameters A and B, together with the thicknesses of the remaining flakes, were refitted using the same normal-incidence TMM and the same AFM thickness bounds as in the baseline analysis. All leave-one-out fits converged successfully for both materials. For h-BN, the largest variation was $|\Delta A| = 0.0135$, $|\Delta B| = 0.003\, \mu m^2$, and max$|\Delta n| = 0.013$. For α-$MoO_3$, the corresponding value were $|\Delta A| = 0.0151$, $|\Delta B| = 0.006\, \mu m^2$, and max$|\Delta n| = 0.013$. Therefore, the leave-one-out analysis indicates that the extracted refractive indices are stable with respect to the calibration set and are not driven by a single outlier flake or spectrum.

Note S6. Determination of flake thicknesses.

After extracting the refractive index dispersion of h-BN and α-MoO_3, the thicknesses of individual flakes were determined by fitting their experimental reflectance spectra to the theoretical reflectance calculated using the isotropic TMM. In these fits, the refractive index of the material was fixed to the Cauchy dispersion obtained from the global calibration described in Note S4, while the flake thickness d was used as the fitting parameter.

For each flake, the residual was defined as

$$r(\lambda) = R_{exp}(\lambda) - R_{TMM}(\lambda, d), \quad (S20)$$

where $R_{exp}(\lambda)$ is the experimental Whittaker-smoothed reflectance spectrum used for fitting and $R_{TMM}(\lambda, d)$ is the calculated reflectance for a trial thickness, The best-fit thickness was obtained by minimizing the sum of squared residuals over the selected wavelength range using scipy.optimize.curve_fit.

To quantify the quality of each thickness fit, several standard goodness-of-fit metrics were calculated from the residuals on the fitting wavelength range, including MSE, RMSE and NRMSE. In addition, the maximum absolute residual was monitored to identify the regions where the disagreement between experiment and theory was largest. Figure S2 shows some representative thickness fits for h-BN and α-MoO_3 flakes used in the validation of the refractive indices in Figure 1g of the main manuscript, together with the corresponding wavelength-dependent residuals.

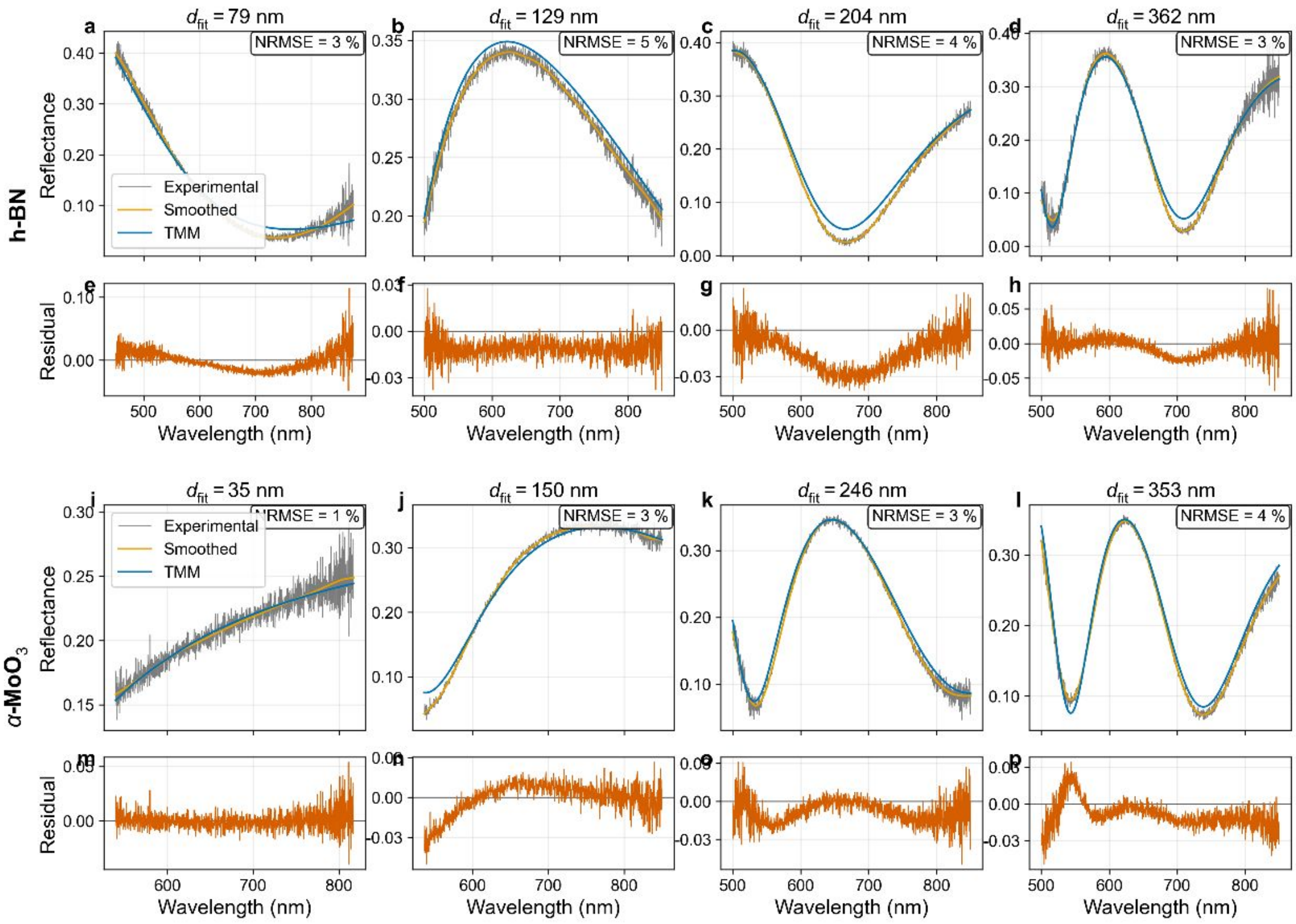

Fig. S2. Representative thickness fits and residuals for h-BN and α-MoO_3 flakes. (a-d) Representative experimental micro-reflectance spectra of h-BN flakes on Si, together with the corresponding TMM fits. (e-h) Relative residuals corresponding to the h-BN fits shown in panels (a-d). (i-p) Same as panels (a-h), but for α-MoO_3 flakes.

Note S7. Visibility and accuracy on the estimation of large nanoscale air gaps.

In general, from an optical point of view, larger air gaps are not expected to be more challenging to detect. On the contrary, increasing the air-gap thickness introduces a larger optical path difference within the multilayer stack and therefore produces larger shifts of the interference maxima and minima in the reflectance spectrum. As a result, the spectral changes associated with a >100 nm air gap should remain clearly detectable. To support this point using the same reflectance-based strategy employed in the manuscript, we performed additional TMM simulations for representative air-gap thicknesses on air/360-nm-thick α-MoO_3 flake/air-gap/Si-substrate multilayer structures extending well beyond the experimentally validated range to address the following points: 1) whether is possible to resolve large air-gaps with arbitrary thicknesses; 2) accuracy on the air-gaps' thicknesses estimation values.

Possibility to resolve large air-gaps with arbitrary thicknesses.
Figure S3 shows TMM-simulated reflectance spectra for air/α-MoO_3/air-gap/Si-substrate multilayer structures with air-gap thicknesses ranging from <100 nm (Figure S3a), and up to 500 nm (Figure S3b). The integrated intensity contrast was calculated over a 730-750 nm spectral window as the area of the absolute difference between the sample and reference reflectance spectra, normalized by the integrated reference reflectance

$$C = \frac{\int |R_{sample}(\lambda) - R_{ref}(\lambda)| d\lambda}{\int R_{ref}(\lambda) d\lambda} \qquad (S21)$$

By integrating the absolute spectral difference, positive and negative variations produced by shifts of interference maxima and minima cannot cancel each other, so the resulting value directly quantifies the magnitude of the optical change within the selected spectral window. The 730-750 nm integration window was chosen because it is centered near a reflectance minimum of the reference spectrum, where small interference-induced spectral shifts produce a stronger intensity variation, as it is in the cases reported in this work.

Our calculations show that all simulated air-gap thicknesses, below the 360-nm-thick α-MoO_3 flake, remain optically visible as shown in Figure S3c, although with different contrast amplitudes. A contrast value of zero would indicate that the sample and reference spectra are indistinguishable within the selected integration window, whereas higher values indicate stronger optical sensitivity. In this normalized representation, a value of one corresponds to an integrated absolute spectral difference equal to the integrated reference reflectance. Finally, it is important to note that, whereas the integrated intensity provides an efficient contrast mechanism for identifying spatial regions with different buried air gaps in the micro-reflectance maps, the exact air-gap thickness can be subsequently obtained by fitting the full reflectance spectrum at each region with the TMM model following the procedure mentioned in the main text of the manuscript.

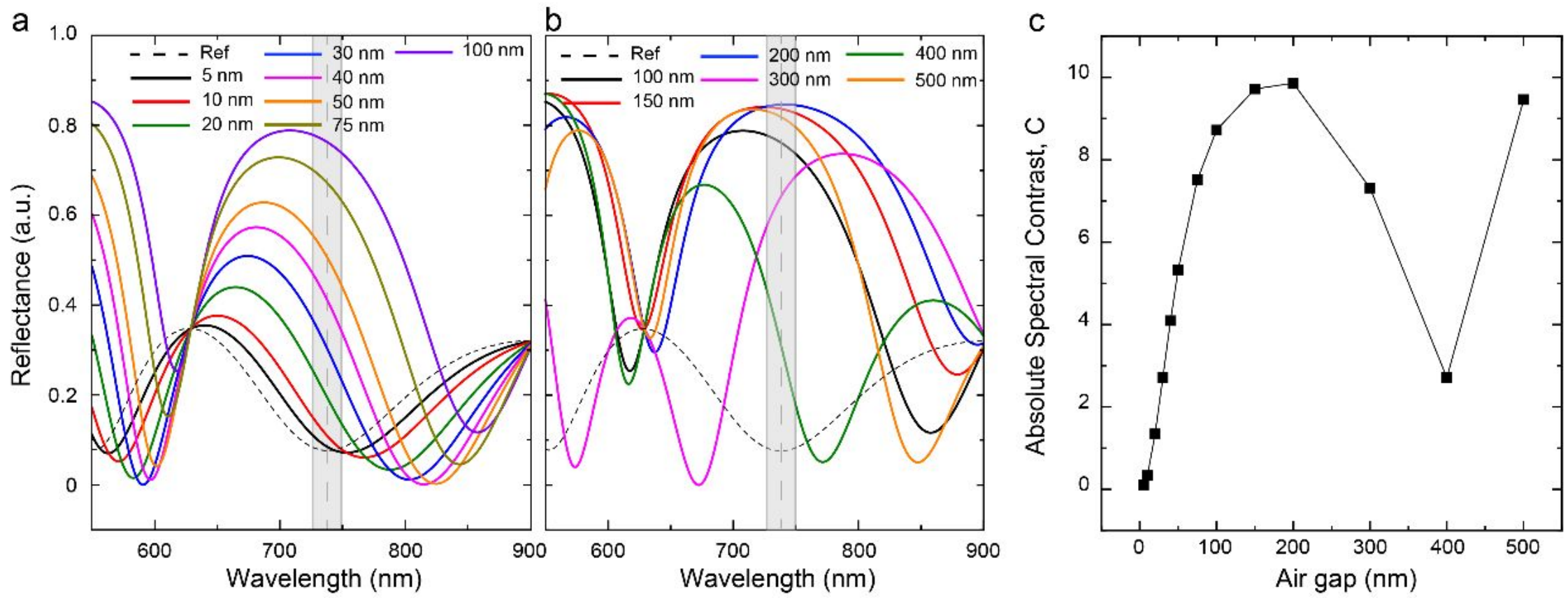

Figure S3. Simulated reflectance spectra for an air/360-nm-thick MoO_3/air-gap/Si-substrate multilayer. Spectra reflectance air-gap thicknesses below (a) and above (b) 100 nm, respectively, where the dashed black curve corresponds to the reference air/360-nm-thick MoO_3/Si-susbtrate multilayer structure. The shaded region indicates the 730-750 nm integration window, chosen near one minimum of the reference spectrum. (c) Absolute spectral contrast integrated over the integration window.

Accuracy on the air-gaps' thicknesses estimation values.

We have added a TMM-based uncertainty analysis to estimate the expected precision of the air-gap retrieval from the reflectance spectra. In particular, we have calculated the spectral sensitivity of the calculated reflectance to changes in air-gap thickness g. To account for the experimental noise of the optical setup, we considered a wavelength-independent reflectance noise with standard deviation σ_R. In our analysis, σ_R corresponds to the experimentally estimated reflectance noise. It was obtained from the residual between the raw reflectance spectrum and the corresponding Whittaker-smoothed spectrum. The noise level was then calculated as the Gaussian-equivalent median absolute deviation of this residual, providing a robust estimate of the standard deviation of the experimental reflectance fluctuations. Under this assumption, the expected uncertainty in the retrieved air-gap thickness , σ_g, can be estimated as

$$\sigma_g(g) = \frac{\sigma_R}{\sqrt{\sum_\lambda \left(\frac{\partial R(\lambda,g)}{\partial g}\right)^2}} = \frac{\sigma_R}{D(g)} \qquad (S22)$$

Here, σ_g is the expected uncertainty in the extracted air-gap thickness, expressed in nm, and σ_R is the standard deviation of the experimental reflectance noise. In this expression, the denominator represents the sensitivity of the calculated reflectance spectrum to changes in the air-gap thickness g. We denote this sensitivity term as $D(g)$. The squared value in the denominator avoids cancellations between wavelengths where the reflectance increases or decreases with g, and weights more strongly the spectral regions that are most sensitive to changes in the air-gap thickness. Therefore, σ_g estimates the uncertainty in the extracted air-gap thickness, where for a given reflectance noise σ_R, the uncertainty in g decreases when the reflectance spectrum changes more strongly with g. Thus, a larger $D(g)$ means that small changes in g produce stronger spectral changes, leading to a smaller uncertainty σ_g.

Our calculations show that the estimated uncertainty in the extracted air-gap thickness depends on both the experimental reflectance noise and the specific multilayer configuration (Figure S4). Specifically, for the representative noise level of our setup, σ_R ~ 0.05, the calculated uncertainty remains on the order of a few nanometres over most of

the simulated air-gap range and for all α-MoO_3 flake's thicknesses, including air gaps above 100 nm. Importantly, in the shallow-gap range experimentally validated in this work, below ~40 nm, the calculated uncertainty remains in the nanometre range, consistent with the excellent agreement obtained between AFM and micro-reflectance for the 7-40 nm trench depths reported in the main manuscript. As expected, the uncertainty increases in regions where the spectral sensitivity $D(g)$ is lower, reflecting the fact that the reflectance spectrum is less sensitive to changes in g under those specific interference conditions. Nevertheless, even in these less favourable regions, the uncertainty remains sufficiently small to distinguish large air gaps with sufficiently enough resolution of about 1-2 nm.

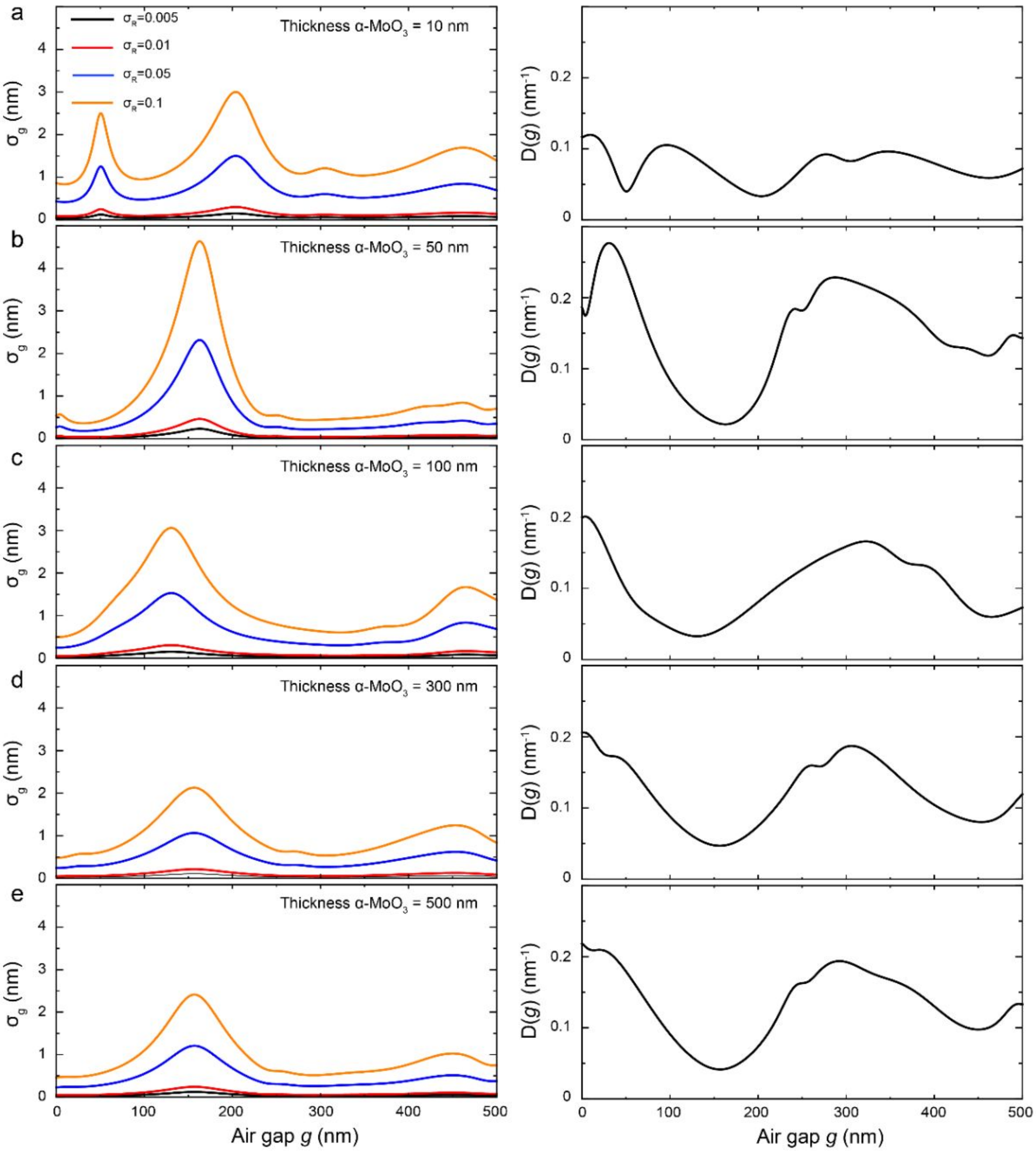

Figure S4. Estimated air-gap-thickness uncertainty as a function of air-gap thickness and α-MoO_3 overlayer thickness. Left panels: calculated uncertainty in the extracted air-gap thickness, σ_g, as a function of air-gap thickness g for α-MoO_3 overlayer thicknesses of (a) 10 nm, (b) 50 nm, (c) 100 nm, (d) 300 nm, and € 500 nm. Different curves correspond to representative reflectance-noise levels, σ_R = 0.005, 0.01, 0.05, and 0.1. Right panels: corresponding spectral sensitivity term, $D(g)$, obtained from the derivative of the TMM-calculated reflectance spectrum with respect to the air-gap thickness. Lower values of σ_g and higher values of $D(g)$ indicate higher sensitivity to changes in the buried air-gap thickness.

Note S8. Simulated air-gap detection sensitivity as a function of air-gap thickness and overlayer thickness.

In this note, we calculated TMM reflectance spectra for air/flake/Si-substrate and air/flake/air-gap/Si-substrate multilayer structures while systematically varying both the flake thickness and the air-gap thickness. In particular, this analysis addresses two related questions: first, whether buried air gaps remain optically visible when the increasing overlayer thickness introduces additional interference oscillations; and second, whether the air-gap thickness can still be quantitatively extracted with high accuracy from the full reflectance spectrum.

Visibility of buried air gaps for varying α-MoO_3 overlayer thicknesses

To evaluate the robustness of the reflectance-mapping approach to resolve buried air gaps against changes in the transparent overlayer thickness, we performed TMM simulations (Figure S5) for air/α-MoO_3/air-gap/Si multilayer structures with a fixed buried air gap of 10 nm (typical value for realistic air gaps in vdW heterostructures) while varying the α-MoO_3 flake thickness (from 0 to 200 nm). For each thickness, we compared the reflectance response with that of the corresponding reference structure without an air gap. Specifically, we calculated the integrated intensity contrast over different spectral windows as the area of the absolute difference between the sample and reference reflectance spectra, normalized by the integrated reference reflectance (Equation S21). By integrating the absolute spectral difference, positive and negative variations produced by shifts of interference maxima and minima cannot cancel each other, so the resulting value directly quantifies the magnitude of the optical change within the selected spectral window.

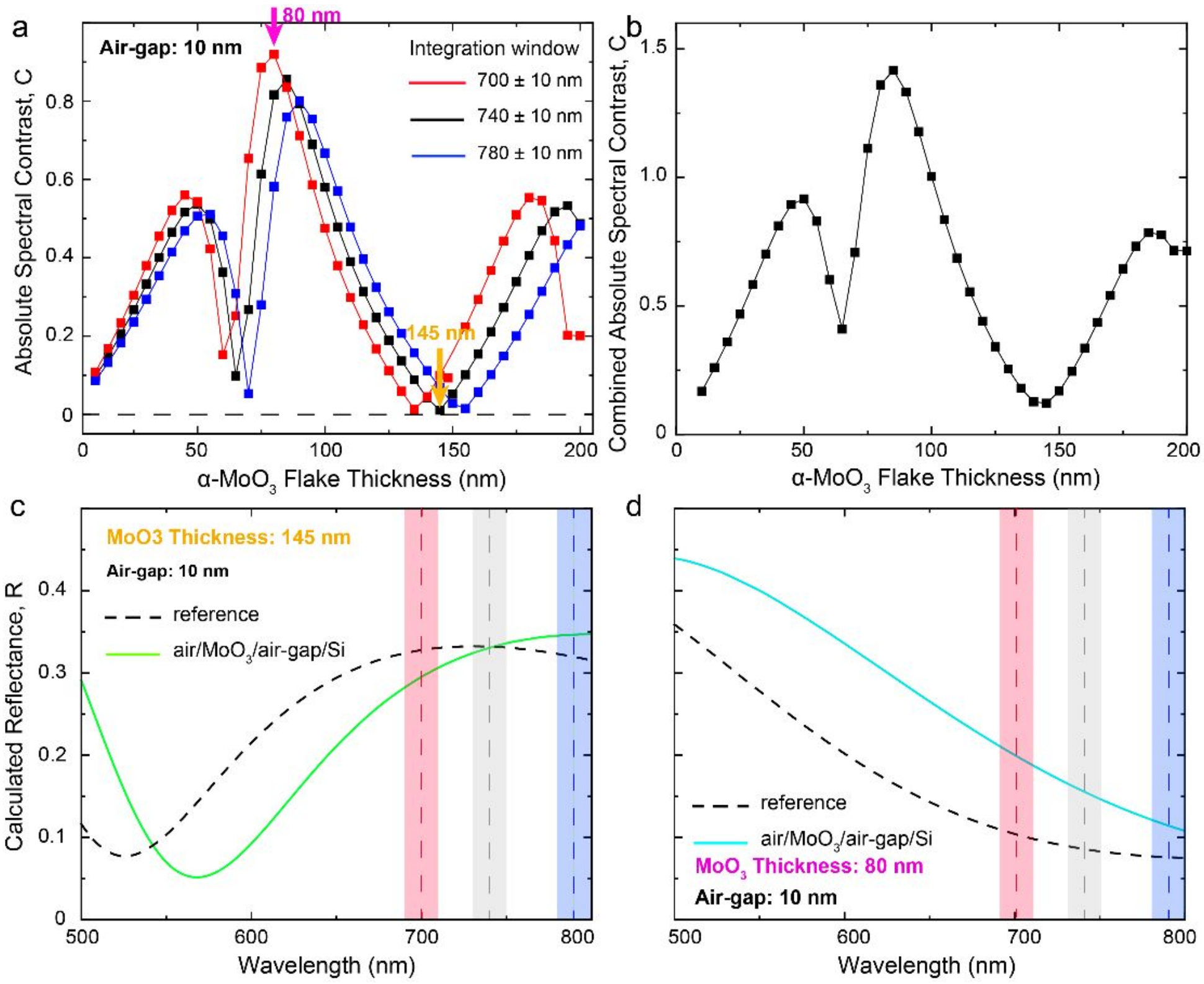

Figure S5. Overlayer-thickness dependence of the reflectance contrast for buried air-gap detection. (a) Simulated absolute integrated-reflectance contrast for an air/α-MoO_3/air-gap/Si multilayer structure with a fixed air-gap thickness of 10 nm, plotted as a function of α-MoO_3 flake thickness. The contrast is calculated with respect to the reference structure without an air gap

using three integration windows centred at 700, 740, and 780 nm, each with a width of ±10 nm. The highlighted regions around 145 nm (orange arrow) and 80 nm (pink arrow) mark representative flake thicknesses for which the contrast in a given spectral window is strongly reduced or enhanced, respectively. (b) Combined integrated-reflectance contrast obtained from the three spectral windows shown in (a) as function of α-MoO_3 thickness. (c,d) Representative TMM-calculated reflectance spectra for α-MoO_3 flakes with thicknesses of (c) 145 nm and (d) 80 nm, with and without (reference) a 10 nm buried air gap, respectively. The shaded regions indicate the three integration windows used in (a).

Our calculations show that the reflectance contrast (i.e., visibility) associated with a buried air gap (air-gap thickness of 10 nm) clearly depends on the α-MoO_3 overlayer thickness, as expected from the thickness-dependent thin-film interference behavior (Figure S5a). It should be noted that, for some specific α-MoO_3 overlayer thicknesses, a single integration window can coincide with a crossing point between the reference spectrum (with no air gap) and the spectrum corresponding to the multilayer structure containing the air gap (see integration window centered at 740 nm in Figure S5c), leading to a strongly reduced contrast in that particular spectral window. In such cases, the air gap could become poorly visible if only one spectral window were used. To avoid this loss of visibility, a multi-window integration strategy can be implemented using three nearby spectral windows. As shown by the combined contrast in Figure S5b, this approach maintains a finite optical contrast over the full range of simulated α-MoO_3 overlayer thicknesses, because a loss of contrast in one window is compensated by finite contrast in the others. The same behavior is also expected to depend on the air-gap thickness, since changing the gap shifts the interference pattern and therefore the spectral regions of maximum sensitivity.
Importantly, this multi-window strategy is mainly relevant when extending the analysis to a broad range of overlayer and air-gap thicknesses. We note that, for the shallow gaps below 50 nm and the flake thicknesses experimentally addressed in the main manuscript, a single integration spectral window already provides sufficient optical contrast, as demonstrated by the experimental maps and by the excellent agreement with AFM.

Accuracy of air-gap thickness extraction as a function of α-MoO_3 overlayer thickness
After evaluating the visibility of the buried air gap, we next assessed the quantitative air-gap-thickness extraction capability as a function of the α-MoO_3 overlayer thickness. For this purpose, we used the full-spectrum TMM sensitivity analysis, evaluating the spectral sensitivity to the air-gap thickness (g) from 0 to 50 nm for different flake's thicknesses. This provides an estimate of the expected uncertainty in the extracted air-gap thickness, σ_g, for each overlayer thickness and for representative experimental reflectance-noise levels. To account for the experimental noise of the optical setup, we considered a wavelength-independent reflectance noise with standard deviation σ_R. In our analysis, σ_R corresponds to the experimentally estimated reflectance noise. It was obtained from the residual between the raw reflectance spectrum and the corresponding Whittaker-smoothed spectrum. The noise level was then calculated as the Gaussian-equivalent median absolute deviation of this residual, providing a robust estimate of the standard deviation of the experimental reflectance fluctuations. Under this assumption, the expected uncertainty in the retrieved air-gap thickness, σ_g, can be estimated using Equation S22.

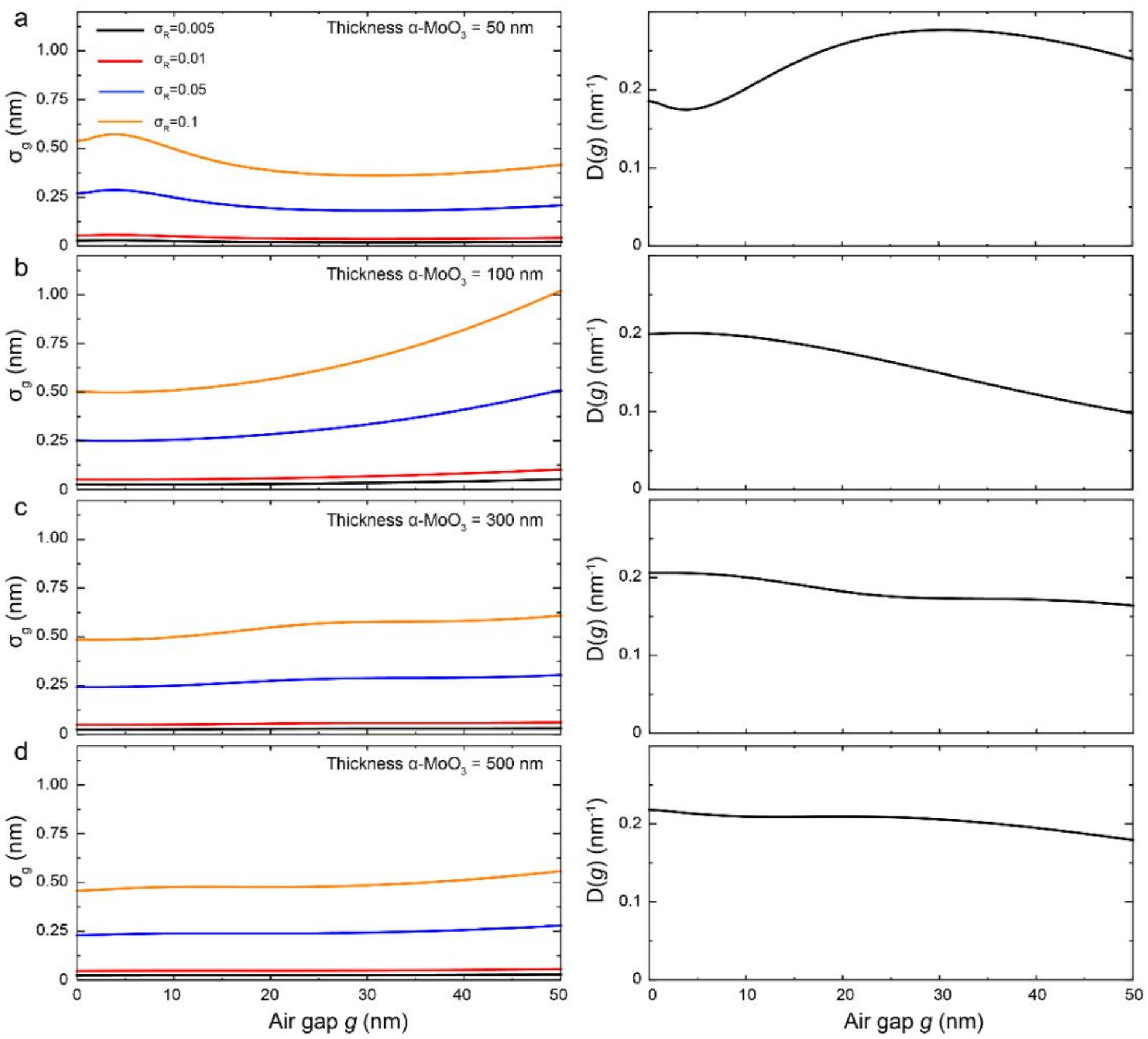

Figure S6. Estimated air-gap-thickness uncertainty as a function of buried gap and α-MoO_3 overlayer thickness. Estimated uncertainty in the extracted air-gap thickness, σ_g, calculated from the full-spectrum TMM sensitivity analysis for air/α-MoO_3/air-gap/Si multilayer structures with α-MoO_3 overlayer thicknesses of (a) 50 nm, (b) 100 nm, (c) 300 nm, and (d) 500 nm. The air-gap thickness g was varied from 0 to 50 nm. Left panels show σ_g for different representative reflectance-noise levels, σ_R = 0.005, 0.01, 0.05, and 0.1. Right panels show the corresponding spectral sensitivity term, $D(g)$, obtained from the derivative of the TMM-calculated reflectance spectrum with respect to the air-gap thickness.

Our calculations in Figure S6 show that, even when the α-MoO_3 overlayer thickness is varied, the expected uncertainty remains well below the air-gap thicknesses considered. For the highest experimental noise level representative of our measurements, (σ_R ~ 0.03), the estimated uncertainty remains below ~ 0.3 nm over most of the analysed cases. This is consistent with the nanometre-scale precision observed experimentally for shallow trenches in the main manuscript. Therefore, the calculations show that although the overlayer thickness modifies the interference pattern and can shift the spectral regions of highest contrast, it does not intrinsically prevent quantitative extraction of the buried air-gap thickness. Instead, the full-spectrum TMM fit remains sensitive to the air-gap over the investigated overlayer thicknesses, which are within the typical range for applications in twisoptics.

Note S9. Sensitivity analysis of the effective-index approximation for biaxial α-MoO_3.

In this note, we evaluate the possible impact of modelling the optical response of biaxial α-MoO_3 using an effective isotropic refractive index, as done in the main analysis. For this purpose, we performed a sensitivity analysis in which reflectance spectra calculated with a full anisotropic 4 × 4 TMM model were used as the reference on [air/α-MoO_3/air-gap/Si-substrate] multilayer structures. These simulated spectra were then fitted with the isotropic 2 × 2 TMM model to quantify the error introduced by the effective-index approximation.

The simulations were performed assuming unpolarized illumination and using representative α-MoO_3 thicknesses of $d_{\alpha-\mathrm{MoO_3}} = 50, 100, 200,$ and 500 nm, combined with air-gap thicknesses of $d_{air} = 0, 5, 10, 20, 30, 40,$ and 50 nm. For each combination of α-MoO_3 and air-gap thickness, the reflectance spectrum was first simulated using the full anisotropic 4 × 4 TMM model. These spectra were then fitted using the simplified isotropic 2 × 2 TMM model with an in-plane averaged effective refractive index,

$$n_{eff} = 0.5 \cdot (n_\alpha + n_\gamma), \qquad (S23)$$

where α and γ correspond to the two in-plane optical axes of α-MoO_3. The refractive indices used for the simulations were the ones reported in Ref.[7]

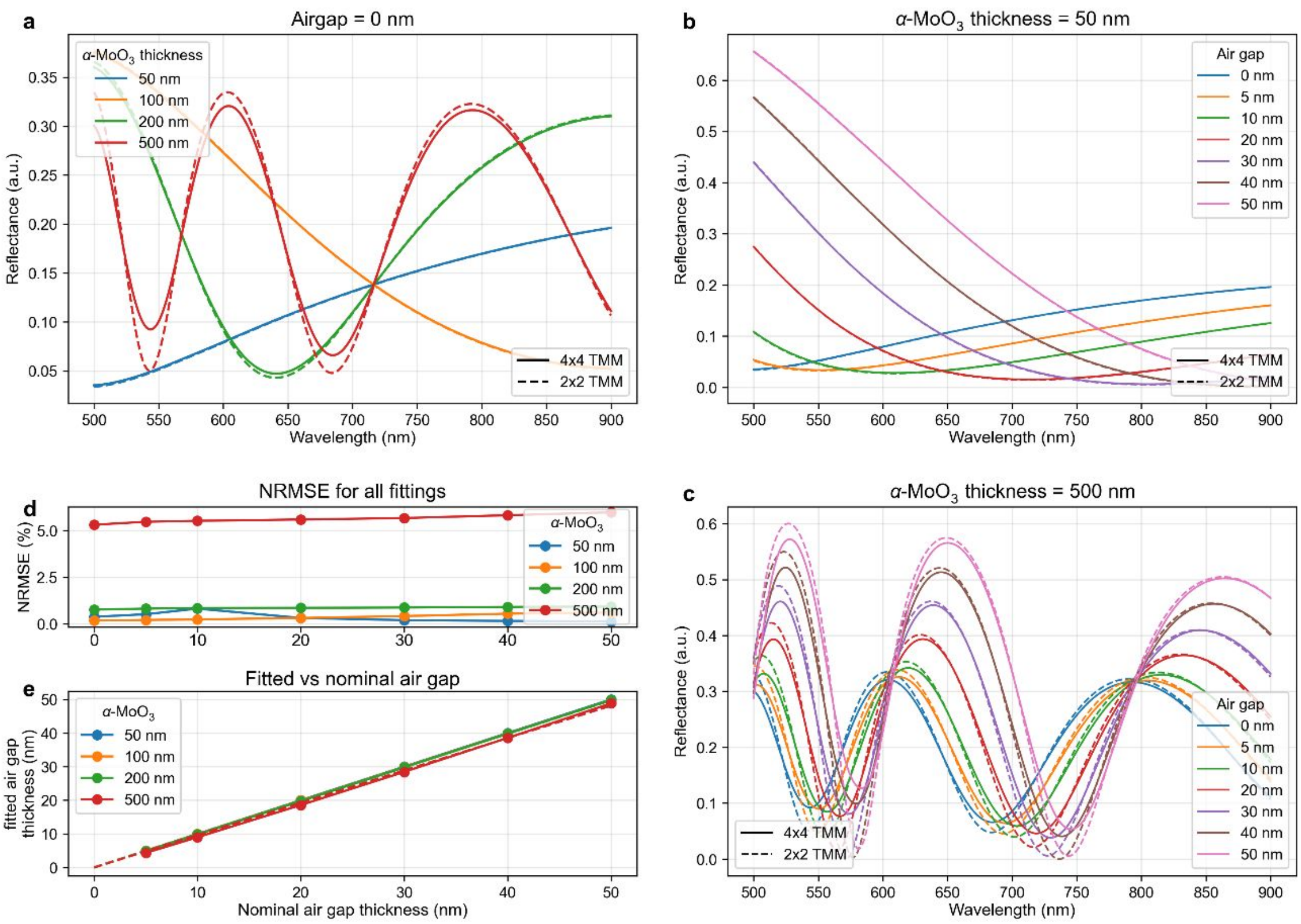

Figure S7. Sensitivity analysis of the effective-index approximation for biaxial α-MoO_3. (a) Calculated **r**eflectance spectra of α-MoO_3 flakes with different thicknesses on a Si substrate, simulated using the full anisotropic 4 × 4 TMM model, and fitted with the isotropic 2 × 2 TMM model using the effective in-plane refractive index (dashed lines). (b, c) Equivalent comparison for fixed α-MoO_3 thicknesses of (b) 50 nm and (c) 500 nm, considering interfacial air gaps of different thicknesses. (d) NRMSE obtained for all fittings. For α-MoO_3 flakes thinner than 200

nm, the NRMSE remains below 1%, while values of approximately 5-6% are obtained for the 500 nm flake. (e) Comparison between the nominal and fitted air-gap thicknesses. The retrieved values remain in close agreement with the nominal ones, with relative errors below 1% for 50-200 nm-thick α-MoO_3 flakes and approximately -4% for the 500 nm-thick flake. In absolute terms, the deviation remains on the order of 1 nm over the studied range.

The comparison between the input values used in the anisotropic 4 × 4 TMM simulations and the values retrieved by the isotropic effective-index 2 × 2 model is shown in Fig. S7. The extracted α-MoO_3 thicknesses and air-gap thicknesses remain in good agreement with the nominal values over the full range considered. In particular, the maximum absolute deviation was 1 nm for both the retrieved α-MoO_3 thickness and the retrieved air-gap thickness.

These results indicate that, under the near-normal-incidence, unpolarized micro-reflectance configuration used in this work, the effective-index approximation does not introduce a significant error in the quantification of either the α-MoO_3 thickness or the interfacial air-gap thickness.

Note S10. Polariton-wavelength sensitivity to nanometric air gaps in α-MoO_3.

To further evaluate the detectability of shallow interfacial gaps, we simulated the polariton wavelength variation in [air/α-MoO_3/air-gap/Si-substrate] multilayers for air-gap thicknesses in the 0-10 nm range. The relative polariton wavelength variation was then defined as

$$\frac{\delta\lambda}{\lambda_0} = \frac{\lambda_p(d_{air}) - \lambda_p(0)}{\lambda_p(0)}\ , \qquad (S24)$$

where $\delta\lambda_p(d_{air})$ is the polariton wavelength for a finite air-gap thickness and $\delta\lambda_p(0)$ is the polariton wavelength in the absence of an air gap.

The calculation was performed for different α-MoO_3 thicknesses and excitation frequencies, since both parameters affect the polariton confinement and therefore the sensitivity to the local dielectric environment. The simulations, shown in Figure S8, indicate that the polariton wavelength shift decreases as the air-gap thickness is reduced. However, this shift also depends strongly on the α-MoO_3 thickness. In thinner flakes, the polariton wavelength is more sensitive to changes in the surrounding dielectric environment, leading to a larger relative wavelength variation for the same air-gap thickness.

For example, for an α-MoO_3 thickness of 50 nm, an air gap as small as 1 nm produces a relative polariton wavelength variation of approximately 5% at 840 cm^{-1} (Figure S8b). In contrast, for thicker α-MoO_3 flakes, the relative variation induced by such small air gaps becomes much weaker over the studied frequency range and may approach the uncertainty associated with extracting the polariton wavelength from the near-field signal.

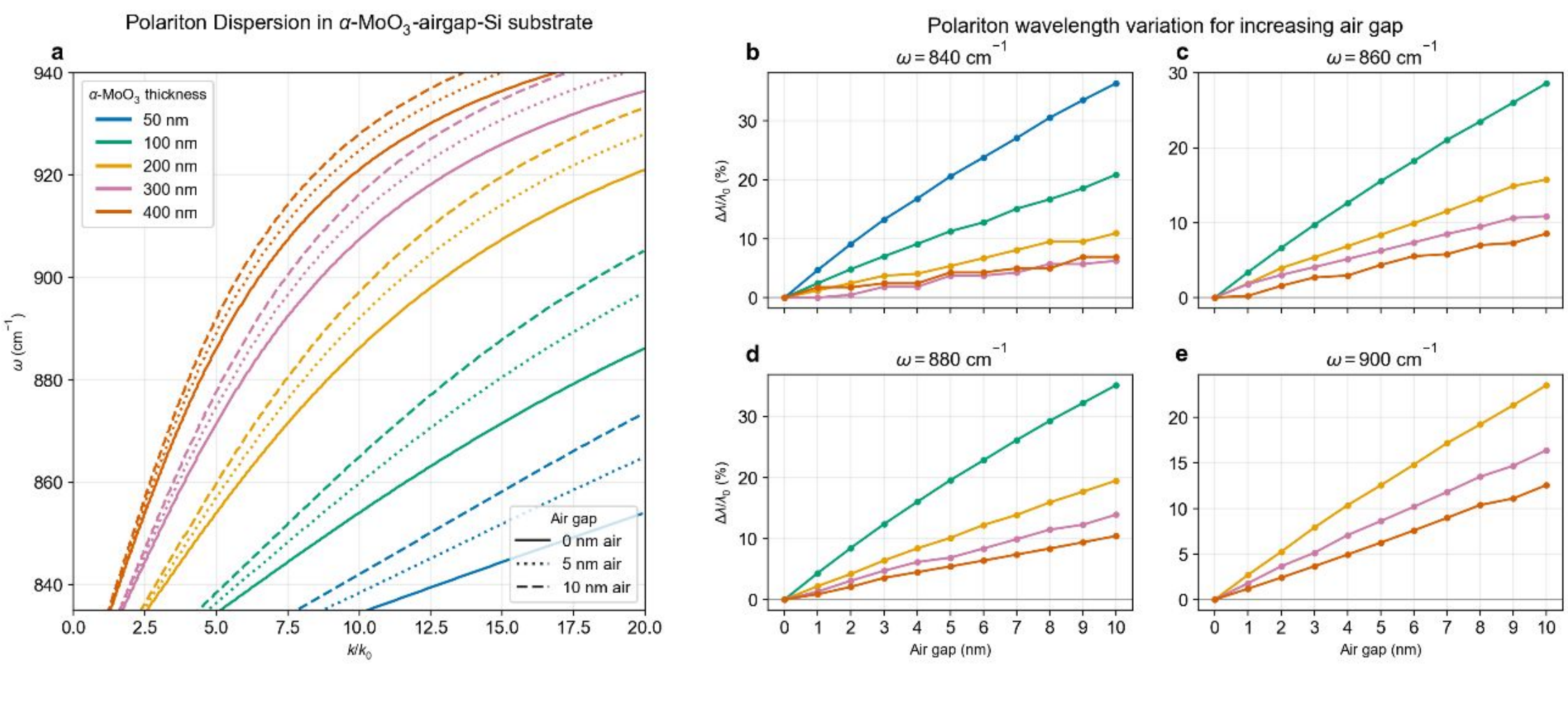

Figure S8. Polariton-wavelength variation induced by nanometric interfacial air gaps. (a) Simulated polariton dispersion for different α-MoO_3 and air gap thicknesses on a Si substrate. (b-e) Relative change in polariton wavelength, $\frac{\delta\lambda_p}{\lambda_p}$, as a function of air-gap thickness for different α-MoO_3 thicknesses at excitation frequencies of (b) 840 cm^{-1}, (c) 860 cm^{-1}, (d) 880 cm^{-1} and (e) 900 cm^{-1}. The relative variation is calculated with respect to the no-air-gap case.

Note S11. Derivation of the dispersion relation of highly confined phonon-polaritons (PhPs) in bilayers of thin film materials.

In this note we show how to obtain the dispersion relation of highly confined polaritons in a twisted bilayer of polar biaxial thin films. Since in the high-momentum approximation the contribution of the ordinary modes can be neglected[8], we only consider *p*-polarized modes for the superstrate and substrate and extraordinary modes for the biaxial layers. Following the same notation as in[8], the boundary conditions for the tangential components of the electric and magnetic fields can be written as follows:

$$\begin{aligned} a_p^{3\uparrow} e^{iq_{3,p,z}(d_1+d_2)} \boldsymbol{p}_3 &= \left(a_{e_2}^{2\uparrow} e^{iq_{2,e,z}(d_1+d_2)} + a_{e_2}^{2\downarrow} e^{-iq_{2,e,z}(d_1+d_2)}\right)\boldsymbol{e}_2 \\ a_p^{3\uparrow} e^{iq_{3,p,z}(d_1+d_2)} \Upsilon_p^3 \boldsymbol{p}_{3,+} &= \left(a_{e_2}^{2\uparrow} e^{iq_{2,e,z}(d_1+d_2)} \boldsymbol{e'}_{2,+} + a_{e_2}^{2\downarrow} e^{-iq_{2,e,z}(d_1+d_2)} \boldsymbol{e'}_{2,-}\right) \end{aligned} \quad \text{(S25)}$$

$$\begin{aligned} \left(a_{e_2}^{2\uparrow} e^{iq_{2,e,z}d_1} + a_{e_2}^{2\downarrow} e^{-iq_{2,e,z}d_1}\right)\boldsymbol{e}_2 &= \left(a_{e_1}^{1\uparrow} e^{iq_{1,e,z}d_1} + a_{e_1}^{1\downarrow} e^{-iq_{1,e,z}d_1}\right)\boldsymbol{e}_1 \\ \left(a_{e_2}^{2\uparrow} e^{iq_{2,e,z}d_1}\boldsymbol{e'}_{2,+} + a_{e_2}^{2\downarrow} e^{-iq_{2,e,z}d_1}\boldsymbol{e'}_{2,-}\right) &= \left(a_{e_1}^{1\uparrow} e^{iq_{1,e,z}d_1}\boldsymbol{e'}_{1,+} + a_{e_1}^{1\downarrow} e^{-iq_{1,e,z}d_1}\boldsymbol{e'}_{1,-}\right) \end{aligned} \quad \text{(S26)}$$

$$\begin{aligned} \left(a_{\gamma_1}^{1\uparrow} + a_{\gamma_1}^{1\downarrow}\right)\boldsymbol{e}_1 &= a_p^{0\downarrow}\boldsymbol{p}_0 \\ a_{\gamma_1}^{1\uparrow}\boldsymbol{\gamma'}_{1,+} + a_{\gamma_1}^{1\downarrow}\boldsymbol{\gamma'}_{1,-} &= a_p^{0\downarrow}\Upsilon_\beta^0 \boldsymbol{p}_0 \end{aligned} \quad \text{(S27)}$$

Eqs. (S25-S27) stand for the boundary conditions at the interfaces between the superstrate (3) and the top (2) layer, between the top (2) and the bottom (1) layers, and between the bottom (1) layer and the substrate (0), respectively. Next, we project Eqs. (S25-S27) onto the subvectors $\boldsymbol{p}$. Considering the approximations $\boldsymbol{p}\cdot\boldsymbol{e}_j = 1$ and $\boldsymbol{p}\cdot\boldsymbol{e'}_{j,\pm} = \frac{\varepsilon_{j,z}q_{j,e,z}}{\pm iq_{j,o,z}^2}$, we rewrite Eqs. (S25-S27) as follows:

$$\begin{aligned} a_p^{3\uparrow} e^{iq_{3,p,z}(d_1+d_2)} &= a_{e_2}^{2\uparrow} e^{iq_{2,e,z}(d_1+d_2)} + a_{e_2}^{2\downarrow} e^{-iq_{2,e,z}(d_1+d_2)} \\ -a_p^{3\uparrow} e^{iq_{3,p,z}(d_1+d_2)} \frac{\varepsilon_3}{iq} &= \frac{\varepsilon_{2,z}q_{2,e,z}}{iq^2}\left(a_{e_2}^{2\uparrow} e^{iq_{2,e,z}(d_1+d_2)} - a_{e_2}^{2\downarrow} e^{-iq_{2,e,z}(d_1+d_2)}\right) \end{aligned} \quad \text{(S28)}$$

$$\begin{aligned} a_{e_2}^{2\uparrow} e^{iq_{2,e,z}d_1} + a_{e_2}^{2\downarrow} e^{-iq_{2,e,z}d_1} &= a_{e_1}^{1\uparrow} e^{iq_{1,e,z}d_1} + a_{e_1}^{1\downarrow} e^{-iq_{1,e,z}d_1} \\ \frac{\varepsilon_{2,z}q_{2,e,z}}{iq^2}\left(a_{e_2}^{2\uparrow} e^{iq_{2,e,z}d_1} - a_{e_2}^{2\downarrow} e^{-iq_{2,e,z}d_1}\right) &= \frac{\varepsilon_{1,z}q_{1,e,z}}{iq^2}\left(a_{e_1}^{1\uparrow} e^{iq_{1,e,z}d_1} - a_{e_1}^{1\downarrow} e^{-iq_{1,e,z}d_1}\right) \end{aligned} \quad \text{(S29)}$$

$$\begin{aligned} a_{\gamma_1}^{1\uparrow} + a_{\gamma_1}^{1\downarrow} &= a_p^{0\downarrow} \\ \frac{\varepsilon_{1,z}q_{1,e,z}}{iq^2}\left(a_{\gamma_1}^{1\uparrow} + a_{\gamma_1}^{1\downarrow}\right) &= a_p^{0\downarrow}\frac{\varepsilon_0}{iq} \end{aligned} \quad \text{(S30)}$$

For convenience, we multiply the second boundary condition in Eqs. (S28-S30) by iq^2, leading to the following system of equations:

$$\begin{aligned} a_p^{3\uparrow} e^{iq_{3,p,z}(d_1+d_2)} &= a_{e_2}^{2\uparrow} e^{iq_{2,e,z}(d_1+d_2)} + a_{e_2}^{2\downarrow} e^{-iq_{2,e,z}(d_1+d_2)} \\ -a_p^{3\uparrow} e^{iq_{3,p,z}(d_1+d_2)}\varepsilon_3 q &= \varepsilon_{2,z}q_{2,e,z}\left(a_{e_2}^{2\uparrow} e^{iq_{2,e,z}(d_1+d_2)} - a_{e_2}^{2\downarrow} e^{-iq_{2,e,z}(d_1+d_2)}\right) \end{aligned} \quad \text{(S31)}$$

$$\begin{aligned} a_{e_2}^{2\uparrow} e^{iq_{2,e,z}d_1} + a_{e_2}^{2\downarrow} e^{-iq_{2,e,z}d_1} &= a_{e_1}^{1\uparrow} e^{iq_{1,e,z}d_1} + a_{e_1}^{1\downarrow} e^{-iq_{1,e,z}d_1} \\ \varepsilon_{2,z}q_{2,e,z}\left(a_{e_2}^{2\uparrow} e^{iq_{2,e,z}d_1} - a_{e_2}^{2\downarrow} e^{-iq_{2,e,z}d_1}\right) &= \varepsilon_{1,z}q_{1,e,z}\left(a_{e_1}^{1\uparrow} e^{iq_{1,e,z}d_1} - a_{e_1}^{1\downarrow} e^{-iq_{1,e,z}d_1}\right) \end{aligned} \quad \text{(S32)}$$

$$\begin{aligned} a_{\gamma_1}^{1\uparrow} + a_{\gamma_1}^{1\downarrow} &= a_p^{0\downarrow} \\ \varepsilon_{1,z}q_{1,e,z}\left(a_{\gamma_1}^{1\uparrow} + a_{\gamma_1}^{1\downarrow}\right) &= a_p^{0\downarrow}\varepsilon_0 q \end{aligned} \quad \text{(S33)}$$

Next, we iteratively solve the system. By dividing the top and bottom boundary conditions in Eq. (S33), we get to

$$a_{\gamma_1}^{1\downarrow} = \left(\frac{\varepsilon_{1,z}q_{1,e,z} - \varepsilon_s q}{\varepsilon_{1,z}q_{1,e,z} + \varepsilon_s q}\right) a_{\gamma_1}^{1\uparrow} \quad \text{(S34)}$$

Inserting Eq. (S34) into Eq. (S32), and dividing the two boundary conditions, we get to

$$\frac{a_{e_2}^{2\uparrow} e^{iq_{2,e,z}d_1} + a_{e_2}^{2\downarrow} e^{-iq_{2,e,z}d_1}}{\varepsilon_{2,z}q_{2,e,z}\left(a_{e_2}^{2\uparrow} e^{iq_{2,e,z}d_1} - a_{e_2}^{2\downarrow} e^{-iq_{2,e,z}d_1}\right)} = D_1 \quad \text{(S35)}$$

where

$$D_1 = \frac{\varepsilon_{1,z}q_{1,e,z}cos(\xi_{1,e,z}) + i\varepsilon_0 q sin(\xi_{1,e,z})}{\varepsilon_{1,z}q_{1,e,z}\varepsilon_0 q cos(\xi_{1,e,z}) + i(\varepsilon_{1,z}q_{1,e,z})^2 sin(\xi_{1,e,z})} \quad \text{(S36)}$$

Eq. (S35) leads to

$$a_{e_2}^{2\downarrow} = e^{2iq_{2,e,z}d_1}\left(\frac{D_1\varepsilon_{2,z}q_{2,e,z} - 1}{D_1\varepsilon_{2,z}q_{2,e,z} + 1}\right) a_{e_2}^{2\uparrow} \quad \text{(S37)}$$

Inserting Eq. (S34) into Eq. (S32), and dividing the two boundary conditions, we get

$$-\frac{1}{\varepsilon_3 q} = \frac{D_1\varepsilon_{2,z}q_{2,e,z}cos(\xi_{2,e,z}) + isin(\xi_{2,e,z})}{\varepsilon_{2,z}q_{2,e,z}cos(\xi_{2,e,z}) + iD_1(\varepsilon_{2,z}q_{2,e,z})^2 sin(\xi_{2,e,z})} \quad \text{(S38)}$$

Eq. (S38) can be rewritten as follows:

$$tan(\xi_{2,e,z}) = \frac{\frac{i}{D_1\varepsilon_{2,z}q_{2,e,z}} + \frac{i\varepsilon_3 q}{\varepsilon_{2,z}q_{2,e,z}}}{1 + \frac{\varepsilon_3 q}{D_1(\varepsilon_{2,z}q_{2,e,z})^2}} \quad \text{(S39)}$$

Using the identity $atan\left(\frac{x+y}{1-xy}\right) = atan(x) + atan(y)$ and noting that $\xi_{2,e,z} = \mp \frac{iqk_0d_2}{\rho_2}$ leads to the following equation describing the dispersion of highly confined polaritons in a twisted bilayer:

$$q = \frac{\rho_2}{k_0 d_2}\left[atan\left(\frac{i\varepsilon_3 q}{\varepsilon_{2,z}q_{2,e,z}}\right) + atan\left(\frac{i}{D_1\varepsilon_{2,z}q_{2,e,z}}\right) + \pi l\right] \quad \text{(S40)}$$

Noting that $q_{j,e,z} = \pm i\frac{q}{\rho_j}$, we can rewrite Eq. (S40) as follows:

$$q = \frac{\rho_2}{k_0 d_2}\left[atan\left(\frac{\varepsilon_3}{\varepsilon_{2,z}}\right) + atan\left(\frac{\rho_2}{D_1\varepsilon_{2,z}}\right) + \pi l\right] \quad \text{(S41)}$$

With

$$\rho_2 = \sqrt{-\frac{\varepsilon_{2,z}}{\varepsilon_{2,x}cos^2(\varphi + \theta_2) + \varepsilon_{2,y}sin^2(\varphi + \theta_2)}} \quad \text{(S42)}$$

$$D_1 = \frac{D_0 \varepsilon_{1,z} q_{1,e,z} cos(\xi_{1,e,z}) + isin(\xi_{1,e,z})}{\varepsilon_{1,z} q_{1,e,z} cos(\xi_{1,e,z}) + D_0 (\varepsilon_{1,z} q_{1,e,z})^2 isin(\xi_{1,e,z})} \text{ (S43)}$$

and

$$D_0 = \frac{i}{\varepsilon_0 q} \text{ (S44)}$$

Note that φ corresponds to the angle with regards to the x axis of our coordinate system and θ_2 to the relative twist angle of the top layer with regards to this axis.

In the case of the samples under study, the top layer is α-MoO_3 and the bottom layer is air. We have taken $\theta_2 = 0$ since we assume the coordinate system to be aligned to the [100] direction of the α-MoO_3 thin film. In addition, $\varepsilon_{2,x}$, $\varepsilon_{2,y}$, and $\varepsilon_{2,z}$ correspond to the permittivity tensor components of α-MoO_3. Since the air layer is isotropic, $\varepsilon_{1,z} = \varepsilon_{air} = 1$ and $q_{1,z} = iq$.

Note 12. Dielectric function of α-MoO_3.

The analytical simulations were performed using the dielectric parameters of α-MoO_3 $\epsilon_x^\infty = 5.92$, $\epsilon_y^\infty = 6.07$ and $\epsilon_z^\infty = 4.47$, and the transverse and longitudinal optical mode frequencies ω_{ij}^{TO} and ω_{ij}^{LO} specified in Table S2 below in Eq. (S41).

main axis i	mode index j	ω_{ij}^{TO} (cm^{-1})	ω_{ij}^{LO} (cm^{-1})
x	1	815	972
x	2	998	1000
y	1	545	850
z	1	962	1007

Table S2. Parameters for the IR dielectric function of α-MoO_3 used for the analytical simulations. The used high frequency permittivities were $\epsilon_x^\infty = 5.92$, $\epsilon_y^\infty = 6.07$ and $\epsilon_z^\infty = 4.47$.

These parameters exhibit slight deviations from previously reported values[9]; although the material is nominally identical, differences in the manufacturer result in minor variations in the dielectric response.

References

(1) Hecht, E. *Optics*, 4th ed.; Pearson Education: San Francisco, CA, 2002.
(2) Gaskell, P. E.; Skulason, H. S.; Rodenchuk, C.; Szkopek, T. Counting Graphene Layers on Glass via Optical Reflection Microscopy. *Applied Physics Letters* **2009**, *94* (14), 143101. https://doi.org/10.1063/1.3115026.
(3) Bing, D.; Wang, Y.; Bai, J.; Du, R.; Wu, G.; Liu, L. Optical Contrast for Identifying the Thickness of Two-Dimensional Materials. *Optics Communications* **2018**, *406*, 128–138. https://doi.org/10.1016/j.optcom.2017.06.012.
(4) Saigal, N.; Mukherjee, A.; Sugunakar, V.; Ghosh, S. Angle of Incidence Averaging in Reflectance Measurements with Optical Microscopes for Studying Layered Two-Dimensional Materials. *Review of Scientific Instruments* **2014**, *85* (7), 073105. https://doi.org/10.1063/1.4889879.
(5) RefractiveIndex.Info: Schinke, Si. https://refractiveindex.info/?shelf=main&book=Si&page=Schinke.
(6) Eilers, P. H. C. A Perfect Smoother. *Anal. Chem.* **2003**, *75* (14), 3631–3636. https://doi.org/10.1021/ac034173t.
(7) Wei, C.; Abedini Dereshgi, S.; Song, X.; Murthy, A.; Dravid, V. P.; Cao, T.; Aydin, K. Polarization Reflector/Color Filter at Visible Frequencies via Anisotropic α-MoO_3. *Advanced Optical Materials* **2020**, *8* (11), 2000088. https://doi.org/10.1002/adom.202000088.
(8) Álvarez-Pérez, G.; Voronin, K. V.; Volkov, V. S.; Alonso-González, P.; Nikitin, A. Y. Analytical Approximations for the Dispersion of Electromagnetic Modes in Slabs of Biaxial Crystals. *Phys. Rev. B* **2019**, *100* (23), 235408. https://doi.org/10.1103/PhysRevB.100.235408.
(9) Álvarez-Pérez, G.; Folland, T. G.; Errea, I.; Taboada-Gutiérrez, J.; Duan, J.; Martín-Sánchez, J.; Tresguerres-Mata, A. I. F.; Matson, J. R.; Bylinkin, A.; He, M.; Ma, W.; Bao, Q.; Martín, J. I.; Caldwell, J. D.; Nikitin, A. Y.; Alonso-González, P. Infrared Permittivity of the Biaxial van Der Waals Semiconductor α-MoO_3 from Near- and Far-Field Correlative Studies. *Advanced Materials* **2020**, *32* (29), 1908176. https://doi.org/10.1002/adma.201908176.
